# Histone Parylation factor 1 contributes to the inhibition of PARP1 by cancer drugs

**DOI:** 10.1038/s41467-021-20998-8

**Published:** 2021-02-02

**Authors:** Johannes Rudolph, Genevieve Roberts, Karolin Luger

**Affiliations:** 1 Department of Biochemistry, University of Colorado Boulder, Boulder, CO 80309 USA; 2 Howard Hughes Medical Institute, Chevy Chase, MD 20815 USA

**Keywords:** Enzyme mechanisms, DNA repair enzymes, Transferases, Target validation

## Abstract

Poly-(ADP-ribose) polymerase 1 and 2 (PARP1 and PARP2) are key enzymes in the DNA damage response. Four different inhibitors (PARPi) are currently in the clinic for treatment of ovarian and breast cancer. Recently, histone PARylation Factor 1 (HPF1) has been shown to play an essential role in the PARP1- and PARP2-dependent poly-(ADP-ribosylation) (PARylation) of histones, by forming a complex with both enzymes and altering their catalytic properties. Given the proximity of HPF1 to the inhibitor binding site both PARPs, we hypothesized that HPF1 may modulate the affinity of inhibitors toward PARP1 and/or PARP2. Here we demonstrate that HPF1 significantly increases the affinity for a PARP1 – DNA complex of some PARPi (i.e., olaparib), but not others (i.e., veliparib). This effect of HPF1 on the binding affinity of Olaparib also holds true for the more physiologically relevant PARP1 – nucleosome complex but does not extend to PARP2. Our results have important implications for the interpretation of PARP inhibition by current PARPi as well as for the design and analysis of the next generation of clinically relevant PARP inhibitors.

## Abstract (web summary)

PARP1 is the target of clinically approved anti-cancer drugs whose in vivo efficacy has been hard to predict. Here the authors show how an altered active site formed between PARP1 and Histone PARylation Factor 1 (HPF1) changes the efficacy of some of these inhibitors.

## Introduction

**Fig. 1:**
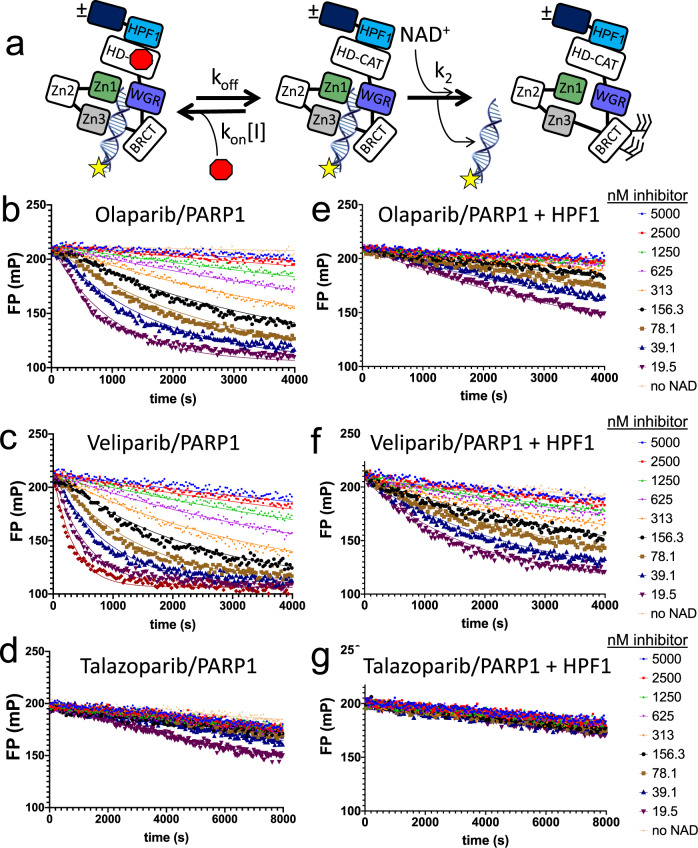
Release of fluorescently labeled DNA from PARP1 reveals binding affinity of PARPi for PARP1 and demonstrates that HPF1 slows the apparent release of PARPi from PARP1. **a** The domains of PARP1 involved in DNA binding (Zn1, Zn2, Zn3, WGR) and the catalytic domain with the HD motif that partially obscures the active site in the absence of DNA are shown as part of the assay scheme. The rates of association and dissociation of the inhibitor are k_on_ and k_off_, respectively. The rate of PARylation of PARP1 is considered constant (k_2_[NAD^+^]) and was determined independently (see Supplementary Fig. 4). **b**–**d** Representative p18mer*-release data for olaparib, veliparib and talazoparib with PARP1 alone. **e**–**g** Representative p18mer*-release data for olaparib, veliparib and talazoparib with PARP1 in the presence of HPF1. The concentrations of the inhibitors used in (**b**–**g**) are noted on the right in units of nanomolar. The lines through the shown data points reflect fitting to first-order kinetics (see Methods).

Poly-(ADP-ribose) polymerase 1 (PARP1), the most abundant nuclear protein after histones^1^, has multiple functions in the nucleus including in the response to DNA damage, chromatin remodeling, RNA transcription, and DNA replication^2–5^. PARP2, a much less abundant protein and the closest homolog to PARP1, collaborates with PARP1 in the response to DNA damage^6^. PARP1 is a multi-domain protein, consisting of four DNA-binding domains (three Zn-fingers and one WGR domain), an automodification domain consisting of a BRCT motif, and a catalytic domain (Fig. 1a). PARP2 has a much smaller N-terminal DNA-binding domain (consisting of an unstructured 76 amino acid region and a WGR domain), while the catalytic domain is very similar to that of PARP1 (46% sequence; 0.55 Å RMSD). Both PARP1 and PARP2 are enzymatically inactive in the absence of DNA. When the DNA-binding domains engage various types of damaged DNA, including single- and double-strand breaks, the HD-motif in the catalytic domain becomes destabilized^7^. Enzymatically active PARP1 and PARP2 result in PARylation, the process of using the substrate NAD^+^ to add long chains of poly-(ADP-ribose) (PAR) onto self (autoPARylation) or other nuclear proteins (transPARylation). The resulting PAR chains recruit DNA repair factors (most notably X-ray repair cross-complementing protein 1 (XRCC) and xeroderma pigmentosum group C-complementing protein) that contain PAR-binding motifs such as macrodomains, WWE-domains, or PAR binding zinc fingers^8^. Deletion of PARP1 leads to increased carcinogenesis, and deletion of both PARP1 and PARP2 is embryonically lethal^9,10^.

PARP1 and PARP2 are recognized as increasingly important targets for cancer therapy with now four clinically approved compounds (olaparib, rucaparib, niraparib, and talazoparib) in use for treatment of breast and ovarian cancer^11^. In addition, these and other inhibitors of PARPs (PARPi) are being investigated as cancer treatments in combination with agents that damage DNA (e.g., temozolomide, cis-platinum, checkpoint inhibitors)^11^. PARPi are also being investigated for applications in treating ischemia-reperfusion injury^12^ and neurodegeneration^13^. All clinically relevant PARPi bind in the NAD^+^ binding pocket of the catalytic domain of PARP1 and PARP2^14^.

The efficacy of PARPi in BRCA1- or BRCA2-deleted cells is based on synthetic lethality^15,16^. Cells treated with PARPi build up stalled replication forks, leading first to single- then double-strand DNA breaks. In normal cells treated with PARPi, DNA repair is mediated by homologous recombination. However, the deficiency of homologous repair in cancerous cells lacking either BRCA1 or BRCA2 leads to cell death^17^. Of additional complication in understanding the cellular effect of PARPi is so-called PARP-trapping, where treatment with PARPi leads to high levels of PARP1 that remain associated with chromatin for extended periods of time^18,19^. The trapping ability of different PARPi does not correlate with potencies measured in vitro but does correlate with their cytotoxicity^18,20,21^. Cells lacking PARP1 have much reduced sensitivity to PARPi, suggesting that PARP-trapping is an important element of the efficacy of PARPi^18,22,23^. Recently, allosteric interactions between PARP1, PARPi, and DNA have been described that further complicate the mechanism by which PARPi can perturb the affinity of PARP1 for DNA^24^.

Histone PARylation factor (HPF1) is a recently discovered essential participant of the physiologically relevant PARylation reaction^25^. As its name implies, HPF1 mediates the transPARylation of histones while also switching the specificity of PARylation from Asp/Glu residues to Ser residues^26,27^. In fact, the Ahel group has recently demonstrated that HPF1 binds to PARP1 and PARP2 and provides the catalytic base (Glu284) that allows PARP1 to perform PARylation on serine residues^28^. The close proximity of HPF1 to the active site of PARP1 and its direct involvement in the chemistry of PARylation raise important questions about the potential role of HPF1 in inhibition by PARPi.

Here we demonstrate that HPF1 significantly increases the affinity of some but not all PARPi for PARP1, but not for PARP2, with the most profound effect for olaparib. We validate and extend these findings by demonstrating that olaparib also increases the affinity of HPF1 for a PARP1—nucleosome complex. Our results have important implications for the interpretation of PARP1 inhibition by current PARPi and will guide the design and analysis of the next generation of PARPi.

## Results

### Sensitive and quantitative assay for determination of K_I_ for inhibitors of PARP1 and PARP2

Determination of accurate binding affinities (K_I_) for potent inhibitors of therapeutic targets is needed to guide drug development but is often a difficult undertaking, in particular for PARP1 and PARP2^29^. Inhibitors of PARPs (PARPi) have been shown to bind with high affinity (pM – nM), using inhibition of enzymatic activity^14,20,30^ or surface plasmon resonance^20,21^ as a readout. Enzymatic assays, especially for PARPs, which are their own preferred substrate, are limited by the minimal amount of enzyme required to detect activity (typically 20–200 nM). In this situation, inhibitor titrations may reflect a titration of active enzyme and do not reveal the true binding constant of the PARPi^31^. Surface plasmon resonance, while possessing greater sensitivity and capable of revealing rates of association and dissociation in addition to K_I_, is often complicated by the flow properties across the surface^32^. Thus, we sought to develop a quantitative solution-based method for accurately determining the true affinity of tight binding inhibitors of PARPs.

We base our method on previous reports that monitored the release of DNA triggered by autoPARylation^18,19,33,34^ and here add the mathematical derivation that allows for the extraction of binding constants (K_I_). Starting with a complex of PARP1 or PARP2 bound to a PARPi and fluorescently labeled DNA, we monitor the loss of fluorescence polarization upon addition of high concentrations of NAD^+^ (2 mM) as the labeled DNA is released from the PARylated PARPs (Fig. 1a). As seen in Fig. 1b for PARP1, there is a strong dependence of the rate of release of labeled DNA on the concentration of olaparib. At the highest olaparib concentrations, virtually no DNA is released, similar to the control without addition of NAD^+^, indicating complete inactivation of PARP1. We see a similar but even more potent effect for the inhibition of PARP2 by olaparib (Supplementary Fig. 1A). When we extend our studies to other inhibitors, we see that veliparib behaves similarly to olaparib for both PARP1 (Fig. 1c) and for PARP2 (Supplementary Fig. 1B). Talazoparib almost completely blocks PARylation for PARP1 and therefore there is little to no DNA release even at the lowest concentrations of inhibitor (Fig. 1d). In contrast, the inhibition of PARP2 by talazoparib is much less dramatic, appearing similar to the inhibition of PARP2 by olaparib (Supplementary Fig. 1C). Representative data for other inhibitors with PARP1 (shown in Supplementary Fig. 2A-D) demonstrate that AZD-2461, niraparib, and A-966492 behave similar to olaparib, while rucaparib appears to be almost as potent as talazoparib. As a control, we found that iniparib, whose reported ability to inhibit PARP1 was not confirmed by later studies^29^, does not have this effect (Supplementary Fig. 3).

**Fig. 2:**
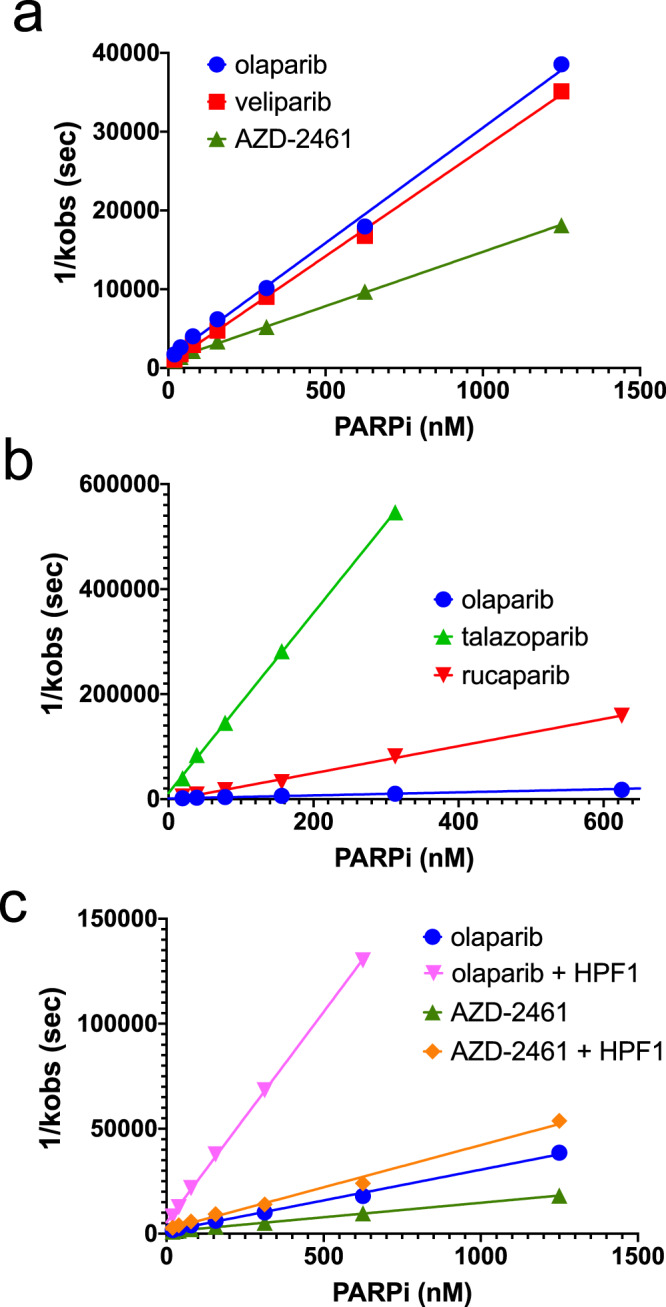
Representative replots for the data collected in Fig. 1 after linearization. **a** The similar potencies for olaparib vs. veliparib are readily apparent from the nearly identical slopes of the lines. In contrast, AZD-2461 is a less potent inhibitor of PARP1. **b** Comparison of the more potent inhibitors talazoparib and rucaparib vs. olaparib. Note the much steeper slopes and change in scale of the *y*-axis compared to (**a**). The data for olaparib in this plot are a duplicate from (**a**) to facilitate direct comparison. **c** The effect of HPF1 on inhibitor potency is readily seen for both olaparib and AZD-2461. The data for olaparib in this plot are a duplicate from (**a**) to facilitate direct comparison. A complete list of derived K_I_ from these replots and multiple replicates are shown in Table 1.

Intuitively one might expect the observed rate of release of labeled DNA to reflect the slow dissociation of PARPi from the complex (k_off_; see Fig. 1a). However, the kinetics are actually more complex as there is competition between the inhibitor re-binding (k_on_[I]) and the binding of, and reaction with, NAD^+^ (k_2_[NAD^+^]) (Fig. 1a). Based on the equations originally used to describe the competition of carbon monoxide for oxygen on hemoglobin^35^, we derive a mathematical model that describes the observed release of DNA (k_obs_, see Methods). To simplify the analysis and to more readily compare different inhibitors, we were able to generate a linearized form of the equation (see Methods) wherein the y-intercept of the line reveals k_off_ and the slope of the line is proportional to 1/K_I_. In practice we find that the intercept is poorly defined since the minimal k_off_, which can be estimated using the limiting assumption of no re-binding of inhibitor (*k*_on_[I] = 0), is quite slow for most inhibitors (*k*_off_ = 0.0001–0.002 s^−1^, *t*_1/2_ = 6–130 min). That is, because only few dissociation events for these tightly binding PARPi occur during the course of a typical 2.5 h experiment, this kinetic parameter remains poorly defined. However, as seen by the dependence of 1/k_obs_ on the concentration of PARPi (Fig. 2a–b), the thermodynamic quantity of K_I_ is well-defined by this experiment. In fact, the replots shown in Fig. 2a–b and used to derive the K_I_ values in Table 1 have *R*^2^ > 0.98.

**Table 1:** K_I_ values (with standard deviations) for PARPi binding to PARP1 and PARP2 alone or in the presence of HPF1.

		PARP1			PARP2		
PARPi	Structure	K_i_ (nM)	K_i_ + HPF1 (nM)	Fold (*t*-test)	K_i_ (nM)	K_i_ + HPF1 (nM)	Fold
Olaparib		0.97 ± 0.17 (*n* = 6)	0.20 ± 0.01 (*n* = 4)	4.8 ***	0.34 ± 0.06 (*n* = 4)	0.39 ± 0.10 (*n* = 4)	0.88 n.s.
AZD-2461		2.2 ± 0.6 (*n* = 6)	0.78 ± 0.14 (*n* = 4)	2.9 **	n.d.	n.d	
A-966492		0.69 ± 0.12 (*n* = 3)	0.32 ± 0.05 (*n* = 3)	2.2 **	n.d.	n.d.	
Niraparib		1.2 ± 0.4 (*n* = 5)	0.64 ± 0.03 (*n* = 3)	1.8 **	n.d.	n.d.	
Veliparib		0.96 ± 0.08 (*n* = 5)	0.78 ± 0.07 (*n* = 4)	1.2 n.s.	9.9 ± 2.8 (*n* = 4)	2.9 ± 2.5 (*n* = 4)	1.7 n.s.
Rucaparib		0.09 ± 0.04 (*n* = 6)	0.07 ± 0.02 (*n* = 5)	1.4 n.s.	n.d.	n.d.	
Talazoparib		0.012 ± 0.003 (*n* = 4)	0.011 ± 0.005 (*n* = 4)	1.1 n.s.	0.18 ± 0.04 (*n* = 3)	0.15 ± 0.03 (*n* = 3)	1.2 n.s.

All values were determined from replots as shown in Fig. 2. The number of replicates for each experiment is indicated, as well as the fold-difference caused by the addition of HPF1. Pairwise comparisons were evaluated using the two-sided student *t* test, as indicated by the asterisks: **P < 0.01, ***P < 0.001.

We find that most of the PARPi we tested have K_I_ in the low single digit nanomolar range for both PARP1 and PARP2 (Table 1) in rough agreement with previous measurements^14,20,30^. The two exceptions for PARP1 are talazoparib and rucaparib, which we find have significantly tighter affinity than previously reported (Fig. 2b, Table 1). The higher affinity of these two compounds was not seen previously using inhibition assays^14,30^ but is in good agreement with BiaCore assays^21^. It is interesting to compare the affinity of olaparib, veliparib and talazoparib between PARP1 and PARP2. While olaparib is somewhat more potent at inhibiting PARP2 compared to PARP1, both veliparib and talazoparib are a full order of magnitude less potent for PARP2 compared to PARP1. The sensitivity of this assay and the analytical tools we provide here will be useful for future quantitative studies of PARPi as they allow for the determination of K_I_ values significantly into the pM range.

As part of our analysis (see Methods), we also derive minimal values of k_off_ (corresponding to maximal half-lives for dissociation; t_1/2_) and minimal values of k_on_ for each inhibitor (Fig. 1a, Supplementary Table 1). We note that olaparib and the other nanomolar binders of PARP1 all have a maximal half-life for dissociation of 5–10 min (Supplementary Table 1). Thus, these inhibitors, while tight-binding, do not have extremely long residence times on PARP1. Rucaparib and talazoparib derive their increased affinity for PARP1 from their very slow dissociation from PARP1, with a maximal half-life for dissociation of 1–2 h. In contrast, the origin of the weaker of affinities of both veliparib and talazoparib for PARP2 compared to PARP1 appear due to their much faster dissociation in comparison to PARP1 (Supplementary Table 1).

### HPF1 increases the affinity of PARPi for PARP1, but not PARP2

Having developed a sensitive assay for the measurement of K_I_ for PARPi binding to PARP1, we next addressed the effect of adding HPF1 to the initial PARP – DNA – inhibitor complex. Although HPF1 does not appear to make any direct contacts with the bound inhibitor EB-47 in the recently solved structure of PARP2 bound to HPF1^28^, its close proximity to the active site suggests that it could have a significant effect on inhibitor binding. As seen by comparing Fig. 1b with 1e, HPF1 has a dramatic effect on the release of labeled DNA from the pre-formed complex of PARP1 – DNA – olaparib – HPF1, which is also clearly seen in the replot in Fig. 2c. The effect of HPF1 is much less pronounced for veliparib (Fig. c vs. f). Given the potency of talazoparib in absence of HPF1, the effect of HPF1 for this compound is difficult to discern (Fig. 1d vs. 1g). Interestingly, and in contrast to PARP1, the effects of adding HPF1 to experiments with PARP2 are much more subtle and go in the opposite direction (Supplementary Fig. 1D-F). That is, the addition of HPF1 allows for apparently faster dissociation of the PARPi, and thus weaker apparent inhibition. Representative data for the remaining inhibitors tested with PARP1 and HPF1 are shown in Supplementary Fig. 2E-H.

Some of the observed slower apparent dissociation of PARPi from PARP1 due to HPF1 can be attributed to slower rates of autoPARylation in the presence of HPF1, since for PARP1, the k_2_[NAD^+^] is 1.4-fold slower compared to controls in the absence of HPF1 (Supplementary Fig. 4A, B). The opposite is true for PARP2, where the apparent more rapid dissociation of PARPi due to HPF1 can be attributed to the faster rates of autoPARylation in the presence of HPF1 as the k_2_[NAD^+^] is 2.7-fold faster compared to controls in the absence of HPF1 (Supplementary Fig. 4C, D). Using our explicit derivation of the individual rate constants, we are able to deconvolute this effect and derive true K_I_ for PARPi binding to PARP1 and PARP2 in the presence of DNA and HPF1 (Table 1). For PARP1, the presence of HPF1 increases the binding affinity for most compounds, especially olaparib (Fig. 2c, Table 1). In contrast, for PARP2 there is no significant effect on the inhibition of olaparib, veliparib, or talazoparib by the addition of HPF1 (Table 1). Confirming that there is a structural aspect with respect to the drug—protein interaction for this effect for PARP1, AZD-2461, a closely related compound to olaparib, shows the second largest difference for its K_I_ in the presence of HPF1 with PARP1 (Fig. 2c, Table 1). Interestingly, the two tightest binding PARPi (rucaparib and talazoparib) did not show any significant changes in their K_I_ in the presence of HPF1. Given the very slow rates of dissociation for these two compounds, we may in part be limited in observing any HPF1 effects by the practicality of performing these experiments for longer time courses. Importantly, we note that for all PARPi evaluated with PARP1, HPF1 decreases the minimal rate of dissociation of the inhibitors significantly, i.e., lengthens the residence time on the enzyme (Supplementary Table 1), suggesting that HPF1’s effect on K_I_ is not caused by slower rates of association for these inhibitors. This slowing of the dissociation of PARPi in the presence of HPF1 is not seen for PARP2, in agreement with their unchanged K_I_ (Supplementary Table).

### PARPi increase the affinity of HPF1 for a PARP1—nucleosome complex

The studies above were performed with free DNA, a much used and validated model for damaged DNA in vitro. However, since HPF1 is known to mediate PARylation of histones, we set out to test whether the effect of HPF1 on the binding of PARPi to PARP1 extends to a more physiological model of damaged DNA, namely nucleosomes. Nucleosomes are the repeating units that form chromatin to organize DNA in eukaryotic cells and consist of a core histone octamer that is wrapped by 147 base pairs of DNA. We used a recombinant nucleosome that has protruding DNA linker ends of 7 and 11 base pairs (Nuc165). Importantly Nuc165 provides both a DNA end for recognition as damaged DNA by PARP1 as well as histone tails with potential PARylation sites that can dock into the groove created at the PARP1 – HPF1 interface^28^. We have previously shown that Nuc165 binds very tightly to PARP1 (K_D_ = 2 nM)^36^.

**Fig. 3:**
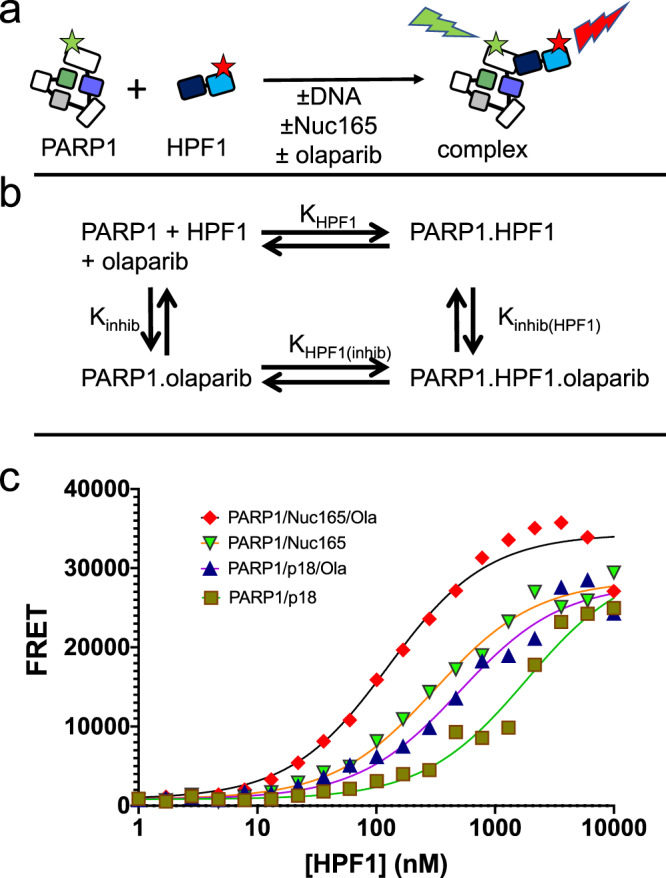
Olaparib increases the effective binding constant of HPF1 for PARP1 in the presence of Nuc165 or p18mer DNA. **a** Schematic for the FRET-based assay utilized to measure the binding of HPF1 to PARP1 in the presence or absence of DNA or nucleosomes. **b** Diagram of the thermodynamic box that allows for interpretation that tighter binding of HPF1 in the presence of olaparib implies tighter binding of olaparib in the presence of HPF1 (K_HPF1_ * K_inhib(HPF1)_ = K_inhib_ * K_HPF1(inhib)_). **c** Representative FRET data demonstrating that the presence of olaparib leads to tighter binding of HPF1 to a PARP1— Nuc165 or a PARP1—p18mer complex. Binding constants, errors, and statistical significance from multiple replicates are enumerated in the text.

Due to the large size of the nucleosome, we could not extend the assay using fluorescence polarization to Nuc165. We therefore developed a FRET-based assay using Alexa488-labeled PARP1 as a fluorescence donor and Alexa647-labeled HPF1 as a fluorescence acceptor to measure the interaction of these two proteins (Fig. 3a). Although this does not provide a direct readout for the affinity of PARPi, we rely on the thermodynamic box principle (Fig. 3b), which states that if HPF1 increases the affinity of PARPi for PARP1, then PARPi must increase the affinity of HPF1 for PARP1. In addition, this assay provides affinity measurements of HPF1 to any PARP1 complex. Because we saw the largest effect of HPF1 using olaparib with PARP1 (Table 1), we focused on this PARPi for these assays.

HPF1 binds to the PARP1—Nuc165 complex with a K_D_ of 790 ± 147 nM (*n* = 12; Fig. 3c). This binding constant becomes 3.5-fold tighter (*p* < 0.0001) in the presence of olaparib (229 ± 55 nM, *n* = 6). Based on the thermodynamic box principle (Fig. 3b), this result implies that HPF1 increases the affinity of olaparib for PARP1 by 3.5-fold when PARP1 is bound to a double-strand break in the context of a nucleosome. This finding of increased affinity for HPF1 in the presence of olaparib holds true when we perform this same FRET experiment using free DNA instead of Nuc165 (Fig. 3c). HPF1 binds to the PARP1 – p18mer complex with a K_D_ of 3800 ± 2600 nM (*n* = 9) and this binding constant becomes 3.2-fold tighter (*p* < 0.004) in the presence of olaparib (1200 ± 500 µM, *n* = 5).

We draw two main conclusions from these results. First, HPF1 binds more tightly to the PARP1—Nuc165 complex than to the PARP1—p18mer complex by a factor of 5 (*p* < 0.0001). This finding helps explain how HPF1, despite its low intranuclear concentration, can co-localize with PARP1 at sites of DNA damage^25^. Presumably the tighter affinity of HPF1 for PARP1 bound to a nucleosome arises in part because HPF1 now has two adjacent binding sites, one for the catalytic domain of PARP1 and the other for a PARylation site on a histone tail of the nucleosome. Second, the 3.2-fold difference for binding of HPF1 in the presence vs. absence of olaparib is in good agreement with the 4.8-fold difference seen for the binding of olaparib in the presence vs. absence of HPF1 (Table 1). Thus, because of the principle of the thermodynamic box, we provide confirmation of the effect of HPF1 on the binding of PARPi for PARP1.

### HPF1 increases the affinity of fluorescent olaparib for PARP1 but not PARP2

**Fig. 4:**
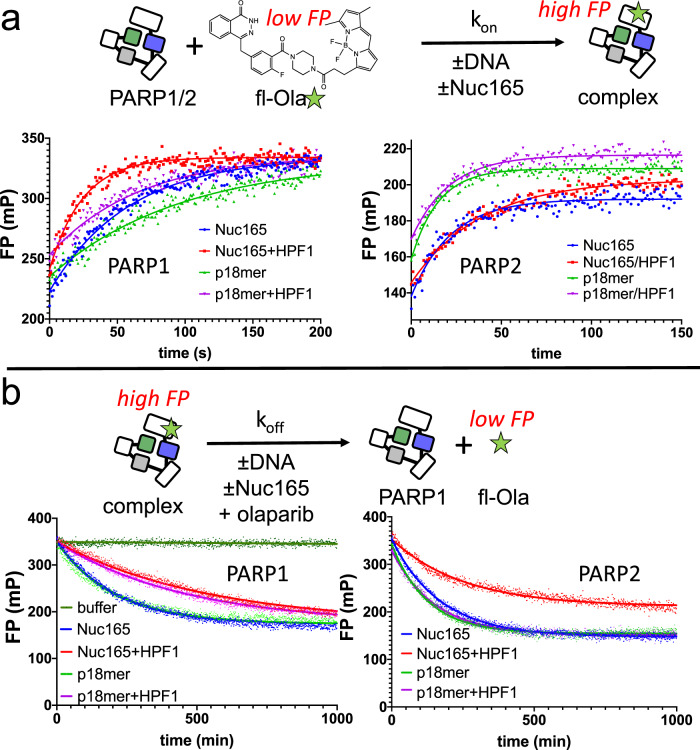
HPF1 increases the affinity of fl-Ola for PARP1 but not PARP2. **a** Top: Schematic for the FP assay used to monitor association of fl-Ola with PARP1 and PARP2. Below: Representative data for monitoring the association of fl-Ola with PARP1 and PARP2 in the presence or absence of p18mer, Nuc165, and/or HPF1. Note the visibly faster association of fl-Ola to PARP1 when HPF1 is present. For PARP2, the association of fl-Ola is slowed in the presence of HPF1. Measured k_on_ from replicates are shown in Table 2. **b** Top: Schematic for the FP assay used to monitor dissociation of fl-Ola from PARP1 and PARP2. Below: Representative data for monitoring the dissociation of fl-Ola from PARP1 and PARP2 in the presence or absence of p18mer, Nuc165, and/or HPF1. Note the visibly slower dissociation of fl-Ola from PARP1 when HPF1 is present. For PARP2, the dissociation of fl-Ola is subtly slowed in the presence of HPF1. Measured k_off_ from replicates are shown in Table 2.

To further validate the effect of HPF1 on the affinity of PARPi for PARP1 but not PARP2, we took advantage of the availability of a fluorescently labeled derivative of olaparib, namely fl-Ola (Fig. 3a). This compound replaces the cyclopropane ring of olaparib with a BODIPY-FL group, has a similar reported binding affinity as olaparib, and is used as an imaging agent to locate glioblastomas in vivo^37^. In order to measure the K_D_ (= k_off_/k_on_) of fl-Ola towards PARP1 and PARP2 in the presence or absence of HPF1, we monitored the rates of association (k_on_) and dissociation (k_off_) by fluorescence polarization (Fig. 3a). We performed this experiment in the presence of either nucleosomes (Nuc165) or free DNA (p18mer) to be able to compare with the results in Fig. 1 and Fig. 3 above. The representative data shown in Fig. 4a (left panel) and summarized in Table 2 reveal that the association of fl-Ola to PARP1 is accelerated in the presence of HPF1 using either p18mer or Nuc165. In contrast, the presence of HPF1 slows the association of fl-Ola with PARP2 (Fig. 4a, right panel; Table 2). The representative data shown in Fig. 4b (left panel) and summarized in Table 2 reveal that the dissociation of fl-Ola (when competed with high concentrations of unlabeled olaparib), is slowed in for PARP1 the presence of HPF1 using either p18mer or Nuc165. For PARP2, as for PARP1, dissociation of fl-Ola is slower in the presence of HPF1 (Fig. 4b, right panel; Table 2). For PARP1, the faster k_on_ and slower k_off_ in the presence of HPF1 yields a 4.4-fold and 2.9-fold tighter K_D_ for fl-Ola in the presence of Nuc165 and p18mer, respectively. These ratios correlate very well with the increased affinity of olaparib as measured in the inhibition assay (Table 1) and the FRET assay (Fig. 3) above. For PARP2, the slightly slower k_on_ and slower k_off_ yield essentially identical K_D_ for all the experimental conditions (Table 2), which correlates well with the lack of an HPF1 effect for the inhibition assays (Table 1). We also note that the true binding affinity of fl-Ola (~10–20 pM) is significantly tighter than previously reported^37^, presumably due to limitations of typical enzyme assays for measuring sub-nM potencies.

**Table 2:** Kinetic and thermodynamic parameters for the binding of fl-Ola to PARP1 and PARP2.

	PARP1			PARP2		
	k_on_ (µM^−1^s^−1^)	k_off_ (hr^−1^)	K_D_ (pM)	k_on_ (µM^−1^s^−1^)	k_off_ (hr^−1^)	K_D_ (pM)
Nuc165	2.2 ± 0.6 *n* = 8	0.27 ± 0.02 *n* = 5	35 ± 11	5.7 ± 1.1 *n* = 4	0.37 ± 0.06 *n* = 4	18 ± 4.6
Nuc165/HPF1	4.4 ± 1.1 *n* = 7	0.12 ± 0.04 *n* = 5	7.9 ± 3.1	2.3 ± 0.2 *n* = 4	0.21 ± 0.03 *n* = 3	24.9 ± 4.7
p18mer	1.3 ± 0.3 *n* = 8	0.32 ± 0.03 *n* = 5	70 ± 18	8.6 ± 2.3 *n* = 3	0.44 ± 0.04 *n* = 3	14.1 ± 3.9
p18mer/HPF1	2.0 ± 0.4 *n* = 7	0.17 ± 0.03 *n* = 5	24 ± 6	5.5 ± 0.8 *n* = 4	0.41 ± 0.08 *n* = 4	20.5 ± 4.8

The values and standard deviations shown are from replicates (n) for each condition performed in duplicate.

## Discussion

Based on the results we present here, it appears that previous screens for inhibitors of PARP1 have been performed in an incomplete and possibly misleading experimental system in that they are missing HPF1, a potentially important contributor to inhibitor potency. We have measured the in-solution binding constants for a variety of clinically relevant inhibitors of PARP1 and PARP2 and have shown that HPF1, a protein known to completely alter the substrate specificity of both PARPs, increases the affinity of some of these inhibitors for PARP1, most significantly for olaparib and its closely related derivative AZD-2461 (Table 1). Interestingly, this HPF1 effect is not seen for PARP2 despite the high similarity of these two proteins in the active site pocket (see below). Although the increases in PARPi affinity are subtle (2 –5 fold), the addition of HPF1 yields effective binding constants that are significantly below the nanomolar range (e.g., 0.2 nM for olaparib). We note that this effect is serendipitous, since to our knowledge, no existing PARPi was developed with the knowledge of a potential role for HPF1. Due to the tight affinity of talazoparib and rucaparib for PARP1 (Table 1), it was not possible to quantitate the effect of HPF1 for these inhibitors using our method. In addition, we find that for PARP1 the presence of HPF1 yields significantly longer half-lives for dissociation for these inhibitors (e.g., 48 min for olaparib compared to 9 min in absence of inhibitor; Supplementary Table 1). Drug efficacy in a clinical setting has been attributed to long-lived protein—inhibitor residence times^38^. We note that our accurate in vitro determinations of K_I_ for different PARPi do not directly inform on the mechanism of PARP-trapping observed in vivo^18^, although our values do lead to a better correlation between strong PARP-trappers (talazoparib and rucaparib), moderate PARP-trappers (olaparib), and weak PARP-trappers (veliparib).

**Fig. 5:**
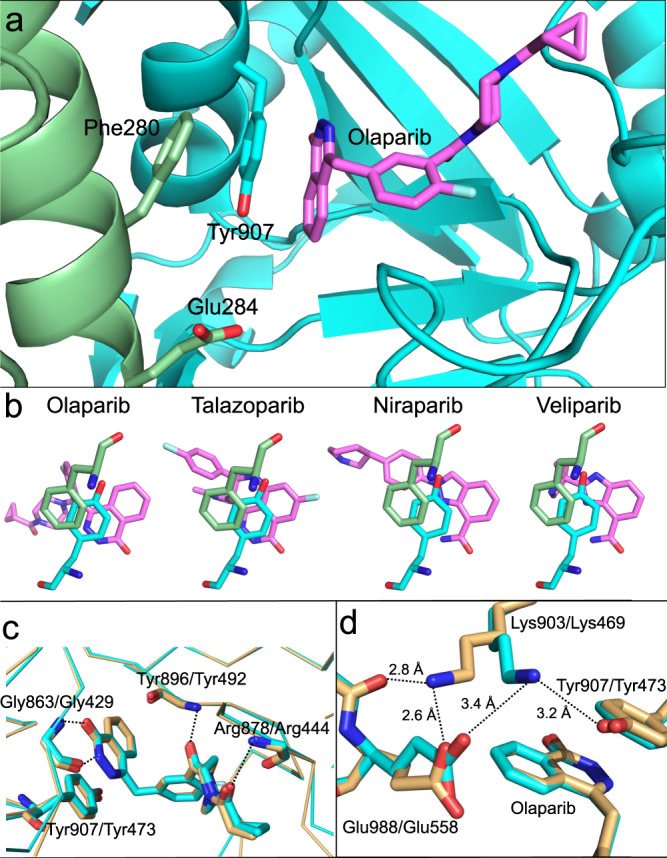
Docking models of the PARP1 – HPF1 – PARPi complexes. **a** Model of the PARP1 active site (in cyan) bound to olaparib (in violet) and docked to HPF1 (in green) showing the pi-pi-pi stack that may explain the increased affinity of olaparib for PARP1 in the presence of HPF1. The catalytic base of HPF1 (Glu284) is the closest residue to olaparib but makes no direct contacts (>6 Å). **b** Olaparib and talazoparib both have pi-pi-pi stacks between Phe280 of HPF1 (in green), Tyr907 of PARP1 (in cyan), and the PARPi (in violet), whereas for niraparib and veliparib this stacking interaction is not as well aligned due to differences in the ring systems. **c** Overlay of the structures of PARP1 (pdb id = 5ds3) and PARP2 (pdb id = 4tvj) both with bound olaparib demonstrating the nearly identical interactions of all active site residues that make direct contact with the inhibitor. The H-bonds shown are all with backbone atoms and the pi-stacking tyrosine is also shown. The backbone trace is shown to emphasize the overall similarity in the catalytic domains (RMSD = 0.55 Å). **d** Representation of the proposed “lysine lock” that may explain why HPF1 affects the inhibition by olaparib of PARP1 (in cyan) but not PARP2 (in light orange).

To understand how HPF1 could be mediating the increased binding affinity of some (but not all) PARPi specifically toward PARP1, we generated models of the PARP1 – HPF1 – PARPi complexes. We began with the published structure of HPF1 docked to the catalytic domain of PARP2 (pdb id = 6tx3) and replaced PARP2 with the very similar catalytic domain of PARP1 in complex with niraparib (pdb id = 4r6e; RMSD = 0.7 Å). Of the many PARP1 – PARPi structures available, we chose the one with niraparib as it has a high resolution (2.2 Å) and most closely mimics the HPF1 – PARP2 interface in 6tx3. In particular, the hydrogen bond between Asp283 of HPF1 and His381 of PARP2^28^, which was shown to be important for the interaction of these two proteins, is conserved using His826 of PARP1. We then used Kcombu^39^ to dock the various PARPi into this model. Notably, in these models none of the PARPi make any direct contact with HPF1. In fact, the closest distance we observe is typically 6–9 Å between the catalytic Glu284 of HPF1 to one of the ring structures of the PARPi (Fig. 4, top). We did note a pi-pi-pi stack involving Phe280 of HPF1, Tyr907 of PARP1, and the aromatic ring structure of olaparib (Fig. 5a, b). This stacking interaction does not occur for niraparib and veliparib, perhaps explaining the smaller effect of HPF1 for these other PARPi (Fig. 5b). We attempted to probe the role of Phe280 of HPF1 with respect to the increase in binding affinity for PARPi in the presence of HPF1. However, the F280A mutant of HPF1 failed to bind the PARP1 – Nuc165 complex (Supplementary Fig. 5a) despite having a similar melting temperature as the wild-type protein (Supplementary Fig. 5B). Thus, Phe280, which is part of a hydrophobic interface with PARP1, appears to be as important as the previously identified hydrogen bond formed using Asp283 for forming a PARP1 - HPF1 complex^28^.

We next searched for the structural basis for why PARP2 does not exhibit an HPF1-mediated increase in the affinity of some PARPi. At first glance, comparisons of the known structures of PARP1 and PARP2 bound to olaparib, veliparib, and talazoparib show essentially identical interactions for each inhibitor within the highly conserved active sites (Fig. 5c). That is, the same direct and/or water-mediated H-bonds are observed between the PARPi and the two PARPs and it is not possible to reconcile the differences in affinities seen for veliparib and talazoparib between the two PARPs. However, we did note a difference in the network of interactions involving the catalytic base (Glu988 in PARP1 and Glu558 in PARP2). In the structure of PARP1 bound to olaparib (pdb id = 5ds3), Glu988 interacts with Lys903, which then interacts with Tyr907 (Fig. 5d), the central member of the pi-pi-pi stack previously discussed (Fig. 4a,b). In the structure of PARP2 bound to Olaparib (pdb id = 4tvj), Glu558 forms a very tight H-bond with the comparable Lys469, which instead of interacting with Tyr473 of the pi-pi-pi stack instead forms an additional tight H-bond with the backbone carbonyl of Asn557 (Fig. 5d). Perhaps the lack of this “lysine lock” for the stacking Tyr473 in PARP2 leads to a less rigid orientation for the tyrosine, thus disrupting the full potential of the pi-pi-pi stack between HPF1, PARP2, and olaparib. These same Glu – Lys configurational differences are seen when comparing veliparib as bound to PARP1 and PARP2 (pdb ids = 2rd6 and 3kjd) but are not relevant to the binding affinities due to the lack of a real pi-pi-pi stack with veliparib. Interestingly, the structures of talazoparib bound to PARP1 and PARP2 (pdb id = 4pjt and 4pjv) both show the PARP2-like configuration.

PARP1 has many roles in the cell in addition to its signaling function in the DNA-damage response, including in transcription, replication, and chromatin remodeling^2–4^. It appears that its function (and the function of PARP2) in DNA-repair is at least in part dependent on HPF1 as loss of HPF1 leads to increased sensitivity to DNA damaging agents^25^. The majority of the other roles of PARP1 may not depend on HPF1, and this is an area of active investigation. Based on our data, we propose that inhibitors should be tailored to the PARP1 – HPF1 pair instead of just PARP1 and may result in higher selectivity for inhibition of PARP1 in the DNA-damage response, and thus for cancer treatment. Presumably this would involve extensive efforts using medicinal chemistry, enzymology, and structural biology that would aim to capture direct HPF1 – PARPi interactions. In contrast, designs of PARPi for applications in non-cancerous settings such as ischemia-reperfusion injury^12^ and neurodegeneration^13^ may want to focus on avoiding this enhanced affinity for the PARP1 – HPF1 complex. Using the methods we have described here, future generations of PARPi can be readily evaluated and steered toward these desired outcomes.

## Methods

### Materials and proteins

Olaparib, AZD-2461, A-966492, niraparib, veliparib, rucaparib, talazoparib, and iniparib were obtained from Selleck and stock solutions (2 mM) were prepared in DMSO. Fluorescent olaparib (fl-Ola) was obtained from Tocris and a stock solution (10 µM) was prepared in DMSO. The p18mer* DNA oligonucleotides and its complementary strand were obtained from IDT: p18mer: 5′-phosphate-5′-fluorescein-GGGTTGCGGCCGCTTGGG-3′. Nucleosome with overhanging ends of 7 and 11 bp was prepared using the Widom601 sequence as previously described^40^. Wild-type PARP1 and PARP2 were expressed and purified as previously described^41,42^. Purification of HPF1 to homogeneity was performed essentially as previously described^25^, with the slight modification of using a gradient for the nickel affinity column from 0 to 40% over 15 CV. The stability of HPF1 was evaluated using the Thermo Fisher Protein Thermal Shift kit according to manufacturer’s instructions.

### Determination of K_I_ for PARPi to PARP1 and PARP2

PARPi (20–5000 nM) diluted in binding buffer (50 mM Tris-HCl (pH 8.0), 50 mM NaCl, 1 mM MgCl_2_, 0.1 mM EDTA, and 0.01% IGEPAL) were titrated across 12 wells of a 384 well plate (Corning 3575) using 1.5-fold dilutions with a final volume of 10 µL. Next, 10 µL of PARP1 or PARP2 (10 nM) premixed with fluorescein labeled p18mer DNA (5 nM), was added to the various dilutions of PARPi and then incubated for 30 min to ensure complete association. Dissociation of labeled DNA was initiated by addition of 10 µL of NAD^+^ (2 mM). All concentrations cited are final concentrations in a volume of 30 µL. When HPF1 was added, it was pre-incubated with DNA and PARPi at a final concentration of 2 µM. Fluorescence polarization (FP) using excitation at 482 nm (bandwidth 16 nm), dichroic filter at 496 nm, and emission at 530 nm (bandwidth 40 nm) was monitored from the top of the plate using a BMG Labtech CLARIOstar plate reader with 30 s intervals over the course of 2.5 h. Kinetic data for a range of different PARPi (20–500 nM) were exported to Prism (GraphPad) and were globally fitted to individual exponentials

1 $$y = \left( {y_0 - y_\infty } \right) \ast e^{\left( { - k_{obs}x} \right)} + y_0$$

with the constraints that y_0_ (the value of the FP signal at *t* = 0) and y_∞_ (the value of the FP signal at *t* = ∞) are shared for all concentrations of PARPi. Under our conditions of high concentrations of NAD^+^, all data are well described by this fitting procedure with *R*^2^ > 0.95, and most typically *R*^2^ > 0.98. (At low concentrations of NAD^+^ we observe significant lag times for the release of DNA that can be adequately modeled by incorporating successive ADPR additions; data and modeling not shown.) Only data that yielded k_obs_ with *R*^2^ > 0.95 were used for further analysis, which lead to frequent exclusion of the data at the highest inhibitor concentrations (1250–5000 nM) as these rates are essentially zero compared to the control in the absence of NAD^+^ and therefore poorly determined. Prior to generating replots of the derived 1/k_obs_ vs. concentration (see derivation below), all rates were corrected for the background loss of FP generated in the same experiment ([PARPi] and [NAD^+^] = 0), which we attribute to slow adherence of protein to the wells of the plate. Accurate determinations of k_2_[NAD^+^] in the presence or absence of HPF1 ([PARPi] = 0; [NAD^+^] = 2 mM), were determined separately under identical experimental conditions using the injector functionality of the plate reader and 1 s read intervals (Supplementary Fig. 4). The measurement of k_2_[NAD^+^] in the presence or absence of HPF1 is essential for elucidating the true K_I_ for the PARPi as seen in the equations below. We emphasize that the same correction is applied to the data for all PARPi. We note that for PARP1, this correction to derive true K_I_ weakens the apparent inhibition, since HPF1-mediated PARylation is slower, and in the case of PARP2 “strengthens” the apparent inhibition, since HPF1-mediated PARylation in this case is faster (see Supplementary Fig. 4). For purposes of deriving an accurate slope of the line in the replot (proportional to 1/K_I_) we estimate a minimal k_off_ from the midpoint of the exponential loss of FP signal (Supplementary Table 2). We can use the K_I_ (derived from the slope of the replot, Fig. 2) and the minimal k_off_ to calculate a minimal k_on_ (K_I_ = k_off_/k_on_, Supplementary Table 2).

**Derivation of equation used to derive K**_**I**_ **for the PARPi:**

$${\mathrm{P}} \cdot {\mathrm{DNA}}^ \ast \cdot {\mathrm{I}}\ \mathop{\leftrightarrows}\limits_{{[{\mathrm{I}}]{\mathrm{k}}_{{\mathrm{on}}}}}^{{k_{{\mathrm{off}}}}}\ {\mathrm{P}} \cdot {\mathrm{DNA}}^ * \mathop{\longrightarrow}\limits^{{[{\mathrm{NAD}}]{\mathrm{k}}_2}}\mathop{\longrightarrow}\limits^{{[{\mathrm{I}}]}}{\mathrm{ppP}} \cdot {\mathrm{I}}$$

Assumptions:

Experiment is performed well above the K_D_ for DNA* (no free DNA*)

Steady-state assumption: $\frac{{d\left[ {P.DNA^ \ast } \right]}}{{dt}} = 0$

Tight-binding assumption for inhibitor $\left( {K_I \ll \left[ P \right]_{total}} \right) \to \left[ P \right]_{total} \gg \left[ {P.DNA^ \ast } \right]$

PARylation rate is saturated: [NAD]k_2_ is independent of [NAD].

PARylation rate is irreversible (no k_−2_).

Binding of I to ppP is kinetically silent as it happens after DNA* release A single PARylation event is sufficient to cause DNA* to dissociate from P note: the fact that this is not true leads to a lag (curvature) at start of reaction note: since curves fit single exponential with *R*^2^ > 0.95, this is a minor effect on fitting. Accounting for species:

2 $$\left[ {P} \right]_{total} = \left[ {P.DNA^ \ast .I} \right] + \left[ {P.DNA^ \ast } \right] + \left[ {ppP.I} \right]$$

3 $$\left[ {P.DNA^ \ast .I} \right]=\left[ P \right]_{total} - \left[ {P.DNA^ \ast } \right] - \left[ {ppP.I} \right]\quad {\mathrm{rearrange}}$$

4 $$\frac{{d\left[ {P.DNA^ \ast .I} \right]}}{{dt}} = - \frac{{d\left[ {P.DNA^ \ast } \right]}}{{dt}} - \frac{{d\left[ {ppP.I} \right]}}{{dt}}\,{\mathrm{follows}}\,{\mathrm{from}}\,{\mathrm{conservation}}\,{\mathrm{of}}\,{\mathrm{P}}_{{\mathrm{total}}}$$

5 $$\frac{{d\left[ {P.DNA^ \ast .I} \right]}}{{dt}} = - \frac{{d\left[ {ppP.I} \right]}}{{dt}}{\mathrm{incorporate}}\,{\mathrm{steady}}\,{\mathrm{state}}\,{\mathrm{assumption}}$$

Rate of interest:

6 $$\frac{{d[ppP.I]}}{{dt}} = \left[ {NAD} \right]k_2 \ast \left[ {P.DNA^ \ast } \right] = - \frac{{d\left[ {P.DNA^ \ast .I} \right]}}{{dt}}\,{\mathrm{from}}\,{\mathrm{above}}$$

Derivation:

$$\frac{{d\left[ {P.DNA^ \ast } \right]}}{{dt}} = \left[ {P.DNA^ \ast .I} \right]k_{off} - \left[ {P.DNA^ \ast } \right]\left[ I \right]k_{on} - \left[ {P.DNA^ \ast } \right]\left[ {NAD} \right]k_2\,{\mathrm{flux}}\,{\mathrm{thru}}\,{\mathrm{pathway}}$$

$$0 = \left[ {P.DNA^ \ast .I} \right]k_{off} - \left[ {P.DNA^ \ast } \right]\left( {[I]k_{on} + \left[ {NAD} \right]k_2} \right)\,{\mathrm{rearrange}}\,{\mathrm{and}}\,{\mathrm{make}}\,{\mathrm{s}}{\mathrm{.s}}.\,{\mathrm{assumption}}$$

$$0 = \left( {[P]_{total} - \left[ {P.DNA^ \ast } \right] - \left[ {ppP.I} \right]} \right)k_{off} - \left[ {P.DNA^ \ast } \right]\left( {[I]k_{on} + \left[ {NAD} \right]k_2} \right)\,{\mathrm{incorporate}}\,{\mathrm{conservation}}\,{\mathrm{of}}\,{\mathrm{P}}.$$

$$\left( {\left[ P \right]_{total} - \left[ {ppP.I} \right]} \right)k_{off} = \left[ {P.DNA^ \ast } \right]\left( {\left[ I \right]k_{on} + \left[ {NAD} \right]k_2 + k_{off}} \right)\,{\mathrm{rearrange}}$$

11 $$\left[ {P.DNA^ \ast } \right] = \frac{{\left( {[P]_{total} - \left[ {ppP.I} \right]} \right)k_{off}}}{{\left[ I \right]k_{on} + \left[ {NAD} \right]k_2 + k_{off}}}\,{\mathrm{rearrange}}$$

12 $$\frac{{d\left[ {ppP.I} \right]}}{{dt}} = \frac{{\left( {[P]_{total} - \left[ {ppP.I} \right]} \right)[NAD]k_2k_{off}}}{{\left[ I \right]k_{on} + \left[ {NAD} \right]k_2 + k_{off}}}{\mathrm{substitute}}\,{\mathrm{from}}\,{\mathrm{rate}}\,{\mathrm{of}}\,{\mathrm{interest}}$$

13 $$\left[ {ppP.I} \right]_{\,} = e^{ - \frac{{[NAD]k_2k_{off}}}{{\left[ I \right]k_{on} + \left[ {NAD} \right]k_2 + k_{off}}}t} + {\it{constant}}\,{\mathrm{integrate}}$$

14 $${\mathbf{ \to }}k_{obs} = \frac{{[NAD]k_2k_{off}}}{{\left[ I \right]k_{on} + \left[ {NAD} \right]k_2 + k_{off}}}$$

Test limits:

If [NAD] = ∞ (or k_2_ = ∞), k_obs_ = k_off_

If [I] = ∞ (or k_on_ = ∞), k_obs_ = 0

If k_off_ = ∞, k_obs_ = [NAD]k_2_

For plotting 1/k_obs_ vs. [I]:

15 $$\frac{1}{{k_{obs}}} = \frac{{\left[ I \right]k_{on} + \left[ {NAD} \right]k_2 + k_{off}}}{{[NAD]k_2k_{off}}}{\mathrm{invert}}$$

16 $$\frac{1}{{k_{obs}}} = \frac{{\left[ I \right]k_{on}}}{{[NAD]k_2k_{off}}} + \frac{1}{{k_{off}}} + \frac{1}{{[NAD]k_2}}{\mathrm{rearrange}}$$

17 $$slope = \frac{{k_{on}}}{{[NAD]k_2k_{off}}} = \frac{1}{{[NAD]k_2K_I}}\left( {{\mathrm{since}}\,{\mathrm{k}}_{{\mathrm{off}}}/{\mathrm{k}}_{{\mathrm{on}}} = {\mathrm{K}}_{\mathrm{I}}} \right)$$

18 $$intercept = \frac{1}{{k_{off}}} + \frac{1}{{[NAD]k_2}} \approx \frac{1}{{k_{off}}}\left( {{\mathrm{since}}\,\left[ {{\mathrm{NAD}}} \right]{\mathrm{k}}_2 \gg {\mathrm{k}}_{{\mathrm{off}}}} \right)$$

### FRET assays to measure affinity o**f** HPF1 for PARP1

The binding of HPF1 to PARP1 in the presence of free DNA or nucleosomes was performed as previously described for PARP2^42^. HPF1 and PARP1 were labeled with Alexa647 C_2_ maleimide and Alexa488 C_4_ maleimide (Invitrogen), respectively, by incubating equimolar ratios of protein with dye for 1 h at 4 °C in 50 mM HEPES (pH 7.5), 150 mM NaCl, 0.1 mM EDTA, and 0.1 mM TCEP. Excess unincorporated dye from HPF1 was removed by repeated dilution and concentration using a centrifugal filter (Millipore 30 kDa MWCO) until no dye absorbance was detectable in the flow through. Excess unincorporated dye from PARP1 was removed by gel filtration chromatography (S-200). The extent of labeling, defined as the ratio of the molar concentration of dye vs. protein, was determined by UV-Vis spectroscopy and was typically 50–100%. HPF1 labeled with Alexa647 was titrated across 20 wells in a 384 well plate (Corning 3575) using 1.5-fold dilutions with a final volume of 10 µL. Next, 10 µL of 488-labeled PARP1 (0.2 µM) premixed with DMSO (control) or olaparib (1 µM), in the presence or absence of p18mer DNA (0.2 µM) or Nuc165 (20 nM) was added to each well. All concentrations reflect final concentrations in the plate and all samples were diluted in binding buffer. The fluorescence intensity was recorded using a BMG Labtech CLARIOstar plate reader at three settings of excitation and emission (noted as excitation wavelength – bandwidth /dichroic wavelength/emission wavelength – bandwidth, all in nm), in this order: 488–20/530/680–50 (FRET channel), 620–30/645/680–50 (acceptor channel), and 488–20/509/535–30 (donor channel). The gain was reset for each experiment, using the well with the maximum acceptor concentration in the donor/acceptor row to set the gain of the FRET channel, using the well with the maximum acceptor concentration in the acceptor/unlabeled row to set the gain of the acceptor channel, and using a well with unlabeled HPF1 in the donor/unlabeled row to set the gain of the donor channel. Raw fluorescence intensity values were used to correct the FRET signal for the donor bleed-through and for the acceptor direct excitation, as previously described^43^. Binding constants (K_d_) were determined by fitting the corrected FRET signal (FRET_corr_)

19 $${{{\mathrm{FRET}}_{{\mathrm{corr}}}}} = {{{\mathrm{FRET}}_{{\mathrm{min}}}}} \\ 	+ \frac{{\left\{ {\left( {{{{\mathrm{FRET}}_{{\mathrm{max}}} - {\mathrm{FRET}}_{{\mathrm{min}}}}}} \right) \ast \left( {\left( {K_d + HPF1 + P1} \right) - \sqrt {\left. {\left( {K_d + HPF1 + P1} \right)^2 - 4 \ast H \ast P1} \right)} } \right)} \right\}}}{{2 \ast P1}}$$

where FRET_max_ is the highest observed FRET signal at saturation, FRET_min_ is the lowest FRET signal in the absence of HPF1, HPF1 is the concentration of HPF1 (0.6–10,000 nM), and P1 is the concentration of the PARP1/p18mer complex (200 nM) or PARP1/nucleosome complex (20 nM).

### Fluorescence polarization assays to measure the binding of fl-Ola to PARP1 and PARP2

Association of fluorescent olaparib (fl-Ola) with PARP1 and PARP2 was determined by diluting fl-Ola (10 nM) in binding buffer for a 30 min pre-incubation in wells of a 384-well plate (Corning 3575) in the presence or absence of DNA (p18mer, 1 µM), Nuc165 (250 nM), and/or HPF1 (2 µM). Next, PARP1 or PARP2 diluted in assay buffer (10 nM) was injected into each well (10 µL) and the FP signal was monitored at 1 s intervals using the settings as described above for the determinations of K_I_. All concentrations reflect final concentrations in the plate. For measurement of dissociation, pre-formed complexes of PARP1 or PARP2 with DNA, Nuc165, and/or HPF1 at the same final concentrations as above during the measurement of association or dissociation were manually mixed with unlabeled olaparib (50 µM) or a mock reaction containing the same final concentration of DMSO (2%). The plates were sealed with a clear film, and the FP signal was monitored every minute for 1000 min. All association and dissociation data were fitted to a single exponential function in Prism (GraphPad) with *R*^2^ > 0.95.

### Reporting summary

Further information on research design is available in the Nature Research Reporting Summary linked to this article.

## Supplementary information

Supplementary Information

Peer Review

Reporting Summary

## Data Availability

The data that support this study is available from the corresponding author upon reasonable request. Source data is provided with this paper.

## Source data

Source Data

## Supplementary information

**Supplementary information** the online version contains supplementary material available at 10.1038/s41467-021-20998-8.

## References

[1] Ludwig A, Behnke B (1988). Immunoquantitation and Size Determination of Intrinsic Poly (ADP-ribose) Polymerase from Acid Precipitates. J. Biol. Chem..

[2] Morales J (2014). Review of poly (ADP-ribose) polymerase (PARP) mechanisms of action and rationale for targeting in cancer and other diseases. Crit. Rev. Eukaryot. Gene Expr..

[3] Ryu KW, Kim DS, Kraus WL (2015). New facets in the regulation of gene expression by ADP-ribosylation and poly(ADP-ribose) polymerases. Chem. Rev..

[4] Ray Chaudhuri A, Nussenzweig A (2017). The multifaceted roles of PARP1 in DNA repair and chromatin remodelling. Nat. Rev. Mol. Cell Biol..

[5] Langelier MF, Eisemann T, Riccio AA, Pascal JM (2018). PARP family enzymes: regulation and catalysis of the poly(ADP-ribose) posttranslational modification. Curr. Opin. Struct. Biol..

[6] Schreiber V (2002). Poly(ADP-ribose) polymerase-2 (PARP-2) is required for efficient base excision DNA repair in association with PARP-1 and XRCC1. J. Biol. Chem..

[7] Dawicki-McKenna JM (2015). PARP-1 Activation Requires Local Unfolding of an Autoinhibitory Domain. Mol. Cell.

[8] Teloni F, Altmeyer M (2016). Survey and summary readers of poly(ADP-ribose): designed to be fit for purpose. Nucleic Acids Res..

[9] Ame JC (1999). PARP-2, A Novel Mammalian DNA Damage-dependent Poly(ADP-ribose) Polymerase. J. Biol. Chem..

[10] Ménissier de Murcia J (2003). Functional interaction between PARP-1 and PARP-2 in chromosome stability and embryonic development in mouse. EMBO J..

[11] Yi M (2019). Advances and perspectives of PARP inhibitors. Exp. Hematol. Oncol..

[12] Berger NA (2018). Opportunities for the repurposing of PARP inhibitors for the therapy of non-oncological diseases. Br. J. Pharmacol..

[13] Kam TI (2018). Poly(ADP-ribose) drives pathologic a-synuclein neurodegeneration in Parkinson’s disease. Science.

[14] Thorsell AG (2017). Structural Basis for Potency and Promiscuity in Poly(ADP-ribose) Polymerase (PARP) and Tankyrase Inhibitors. J. Med. Chem..

[15] Bryant HE (2005). Specific killing of BRCA2-deficient tumours with inhibitors of poly(ADP-ribose) polymerase. Nature.

[16] Farmer H (2005). Targeting the DNA repair defect in BRCA mutant cells as a therapeutic strategy. Nature.

[17] Patel AG, Sarkaria JN, Kaufmann SH (2011). Nonhomologous end joining drives poly (ADP-ribose) polymerase (PARP) inhibitor lethality in homologous. Proc. Natl Acad. Sci..

[18] Murai J (2012). Trapping of PARP1 and PARP2 by clinical PARP inhibitors. Cancer Res..

[19] Murai J (2014). Stereospecific PARP trapping by BMN 673 and comparison with olaparib and rucaparib. Mol. Cancer Ther..

[20] Hopkins TA (2015). Mechanistic Dissection of PARP1 Trapping and the Impact on In Vivo Tolerability and Efficacy of PARP Inhibitors. Mol. Cancer Res..

[21] Hopkins TA (2019). PARP1 trapping by PARP inhibitors drives cytotoxicity in both cancer cells and healthy bone marrow. Mol. Cancer Res..

[22] Shao Z (2020). Clinical PARP inhibitors do not abrogate PARP1 exchange at DNA damage sites in vivo. Nucleic Acids Res..

[23] Juhász S (2020). The chromatin remodeler ALC1 underlies resistance to PARP inhibitor treatment. Sci. Adv..

[24] Zandarashvili L (2020). Structural basis for allosteric PARP-1 retention on DNA breaks. Sci. (80-.)..

[25] Gibbs-Seymour I, Fontana P, Rack JGM, Ahel I (2016). HPF1/C4orf27 Is a PARP-1-Interacting Protein that Regulates PARP-1 ADP-Ribosylation Activity. Mol. Cell.

[26] Bonfiglio JJ (2017). Serine ADP-Ribosylation Depends on HPF1. Mol. Cell.

[27] Palazzo L (2018). Serine is the major residue for ADP- ribosylation upon DNA damage. Elife.

[28] Suskiewicz MJ (2020). HPF1 completes the PARP active site for DNA damage-induced ADP-ribosylation. Nature.

[29] Sinha G (2014). Downfall of iniparib: a PARP inhibitor that doesn’t inhibit PARP after all. J. Natl Cancer Inst..

[30] Shen Y (2013). BMN 673, a Novel and Highly Potent PARP1/2 Inhibitor for the Treatment of Human Cancers with DNA Repair Deficiency. Clin. Cancer Res..

[31] Copeland, R. A. *Evaluation of Enzyme Inhibitors in Drug Discovery: a Guide for Medicinal Chemists and Pharmacologists.* (Wiley, 2013), 978-1118488133. 16350889

[32] Goldstein B, Coombs D, He X, Pineda AR, Wofsy C (1999). The influence of transport on the kinetics of binding to surface receptors: Application to cells and BIAcore. J. Mol. Recognit..

[33] Kurgina TA, Anarbaev RO, Sukhanova MV, Lavrik OI (2018). A rapid fluorescent method for the real-time measurement of poly(ADP-ribose) polymerase 1 activity. Anal. Biochem..

[34] Chen HD (2019). Increased PARP1-DNA binding due to autoPARylation inhibition of PARP1 on DNA rather than PARP1-DNA trapping is correlated with PARP1 inhibitor’s cytotoxicity. Int. J. Cancer.

[35] Gibson QH, Roughton FJ (1955). The kinetics of dissociation of the first oxygen molecule from fully saturated oxyhaemoglobin in sheep blood solutions. Proc. R. Soc. Lond. B. Biol. Sci..

[36] Clark NJ, Kramer M, Muthurajan UM, Luger K (2012). Alternative modes of binding of poly(ADP-ribose) polymerase 1 to free DNA and nucleosomes. J. Biol. Chem..

[37] Irwin CP (2014). PARPi-FL - A Fluorescent PARP1 Inhibitor for Glioblastoma Imaging. Neoplasia.

[38] Copeland RA, Pompliano DL, Meek TD (2006). Drug-target residence time and its implications for lead optimization. Nat. Rev. Drug Discov..

[39] Kawabata T, Nakamura H (2014). 3D flexible alignment using 2D maximum common substructure: Dependence of prediction accuracy on target-reference chemical similarity. J. Chem. Inf. Model..

[40] Muthurajan U (2016). In Vitro Chromatin Assembly: strategies and Quality Control. Methods Enzymol..

[41] Rudolph J, Mahadevan J, Dyer PN, Luger K (2018). Poly(ADP-ribose) polymerase 1 Searches DNA via a ‘Monkey Bar’ Mechanism. Elife.

[42] Gaullier, G. et al. Bridging of nucleosome-proximal DNA double-strand breaks by PARP2 enhances its interaction with HPF1. PLoS ONE. 1–29, 10.1101/846618 (2020). 10.1371/journal.pone.0240932 PMC7608914 33141820

[43] Hieb AR, D’Arcy S, Kramer MA, White AE, Luger K (2012). Fluorescence strategies for high-throughput quantification of protein interactions. Nuc. Acids Res..

