# Supplementary material for: Histone Parylation factor 1 contributes to the inhibition of PARP1 by cancer drugs
Source: Nat Commun. 2021 Feb 2;12:736. doi: 10.1038/s41467-021-20998-8 (PMC7854685; doi:10.1038/s41467-021-20998-8)
Supplement: Supplementary file 1 — Supplementary Information [file 41467_2021_20998_MOESM1_ESM.pdf]

Supplementary Material for

Histone PARylation Factor 1 Contributes to the Inhibition of PARP1 by Cancer Drugs

Johannes Rudolph[1], Genevieve Roberts[1], Karolin Luger*[1,2]

Supplementary Table 1: Maximal $t_{1/2}$ values and minimal k_{on} values (with standard deviation) are shown for each PARPi in the presence of absence of HPF1 for both PARP1 and PARP2. The maximal $t_{1/2}$ values reflect the longest possible half-life for dissociation of different PARPi's from PARP1 (assuming k_{on} = 0). These values were derived from the k_{obs} fits at 19 nM of each PARPi (just over one equivalent PARPi vs. PARP1 or PARP2). The minimal k_{on} values are derived from the measured K_D (see Table 1) and the maximal $t_{1/2}$ values ($k_{on} = \ln2/(K_D{*}\ t_{1/2})$). The number of replicates for each experiment is the same as in Table 1.

Supplementary Figure 1: Release of fluorescently labeled DNA from PARP2 reveals the binding affinity of PARPi for PARP2 and demonstrates that HPF1 does not affect the apparent release of PARPi from PARP2. A) – C) Representative data p18mer*-release data for assay of olaparib, veliparib, and talazoparib with PARP2 alone. D) – F) Representative p18mer*-release data for assay of olaparib, veliparib, and talazoparib with PARP2 in the presence of HPF1. The concentrations of the inhibitors used in A – F are noted on the right in units of nanomolar. The lines through the shown data points reflect fitting to first-order kinetics (see Methods).

Supplementary Figure 2: Release of fluorescently labeled DNA from PARP1 reveals the binding affinity of PARPi for PARP1 and demonstrates that HPF1 slows the apparent release of PARPi from PARP1. A) – D) Representative data p18mer*-release data for assay of AZD-2461, niraparib, A-966492, and rucaparib with PARP1 alone. E) – H) Representative p18mer*-release data for assay of AZD-2461, niraparib, A-966492, and rucaparib with PARP1 in the presence of HPF1. The concentrations of the inhibitors used in A – H are noted on the right in units of nanomolar. The lines through the shown data points reflect fitting to first-order kinetics (see Methods).

Supplementary Figure 3: Release of fluorescently labeled DNA from PARP1 reveals that iniparib is not an inhibitor of PARP1. Note that all concentrations of inhibitor yield the same apparent rate, unlike what is seen for effective PARPi (compare with Fig. 1 and Supp. Fig. 2)

Supplementary Figure 4: Release of fluorescently labeled DNA from PARP1 and PARP2 in the absence of PARPi was measured in the presence or absence of HPF1 to determine the value of $k_{2[}NAD^{+}]$ needed for analysis of the data (see Methods). A) Representative traces from a dissociation experiment from PARP1 performed in the absence or presence of HPF1, without any PARPi present. B) Bar graph from multiple replicates (n>20) demonstrating that PARylation of PARP1 in the presence of HPF1 is 1.4-fold slower than in its absence ($k_{obs(+HPF1)}$ = 0.025 ± 0.006 s^{-1} vs. $k_{obs(-HPF1)}$ = 0.036 ±

0.01 s^{-1}). C) Representative traces from a dissociation experiment from PARP2 performed in the absence or presence of HPF1, without any PARPi present. B) Bar graph from multiple replicates (n>20) demonstrating that PARylation of PARP2 in the presence of HPF1 is 1.4-fold slower than in its absence ($k_{obs(+HPF1)}$ = 0.090 ± 0.011 s^{-1} vs. $k_{obs(-HPF1)}$ = 0.034 ± 0.003 s^{-1}). Note that k_{obs} is the same for both PARP1 and PARP2 in the absence of HPF1.

Supplementary Figure 5: The F280A mutant of HPF1 does not bind to the PARP1 – Nuc165 complex, despite having a similar melting temperature as wild-type HPF1. A) Representative FRET binding experiment performed as in Fig. 3 comparing wild-type HPF1 with the F280A mutant. B) Representative derivative data from the thermal melt curves comparing wild-type HPF1 with the F280A mutant. The average melting temperatures from multiple replicates were 50.6°C ± 0.2°C (n=5) and 51.2°C ± 0.2°C (n=3) for the wild-type and mutant proteins, respectively.

	PARP1				PARP2			
PARPi	$t_{1/2}$ (s)	$t_{1/2}$ (s) + HPF1	k_{on} ($\mu M^{-1}s^{-1}$)	k_{on} ($\mu M^{-1}s^{-1}$) + HPF1	$t_{1/2}$ (s)	$t_{1/2}$ (s) + HPF1	k_{on} ($\mu M^{-1}s^{-1}$)	k_{on} ($\mu M^{-1}s^{-1}$) + HPF1
Olaparib	561 ± 204	2880 ± 887	1.3 ± 0.4	1.2 ± 0.3	245 ± 66	243 ± 43	8.9 ± 2.0	8.2 ± 3.1
AZD-2461	283 ± 120	1245 ± 341	1.3 ± 0.5	0.7 ± 0.1	n.d.	n.d.	n.d	n.d.
A-966492	275 ± 9	2310 ± 52	3.7 ± 0.8	1.0 ± 0.1	n.d.	n.d.	n.d.	n.d.
Niraparib	522 ± 125	1700 ± 35	1.2 ± 0.2	0.6 ± 0.04	n.d.	n.d.	n.d.	n.d.
Veliparib	372 ± 70	1800 ± 594	2.0 ± 0.3	0.5 ± 0.2	83 ± 15	~30	1.0 ± 0.4	4.4 ± 1.6
Rucaparib	3450 ± 1180	>15000	2.8 ± 1.9	0.8 ± 0.2	n.d.	n.d.	n.d.	n.d.
Talazoparib	7300 ± 1200	>20000	8.7 ± 2.9	3.7 ± 1.4	410 ± 35	550 ± 142	9.6 ± 1.8	8.6 ± 1.2

Supp. Table 1

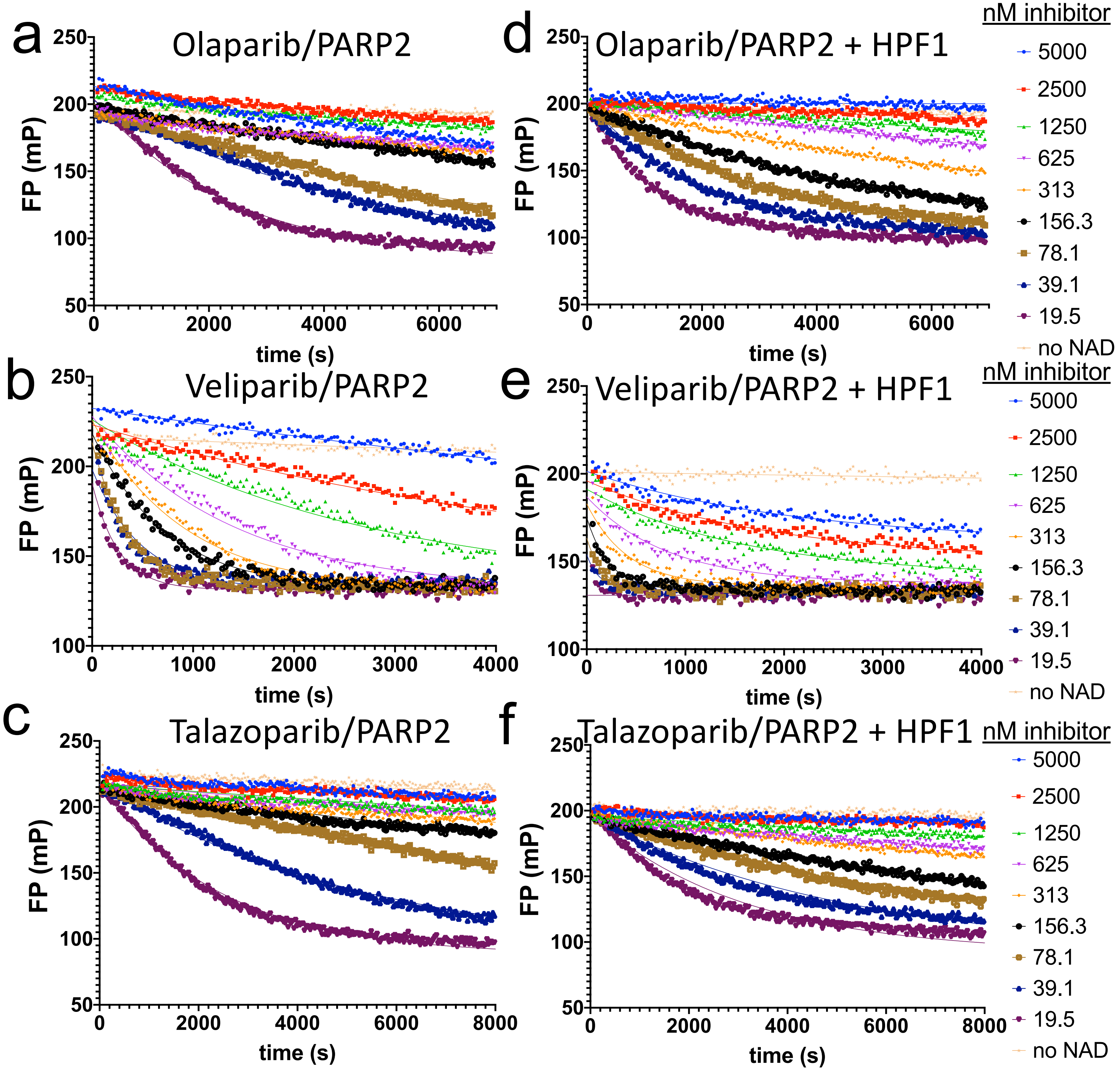

a
Olaparib/PARP2
FP (mP)
time (s)
b
Veliparib/PARP2
FP (mP)
time (s)
c
Talazoparib/PARP2
FP (mP)
time (s)
d
Olaparib/PARP2 + HPF1
FP (mP)
time (s)
nM inhibitor
5000
2500
1250
625
313
156.3
78.1
39.1
19.5
no NAD
e
Veliparib/PARP2 + HPF1
FP (mP)
time (s)
nM inhibitor
5000
2500
1250
625
313
156.3
78.1
39.1
19.5
no NAD
f
Talazoparib/PARP2 + HPF1
FP (mP)
time (s)
nM inhibitor
5000
2500
1250
625
313
156.3
78.1
39.1
19.5
no NAD

Supp. Fig. 1

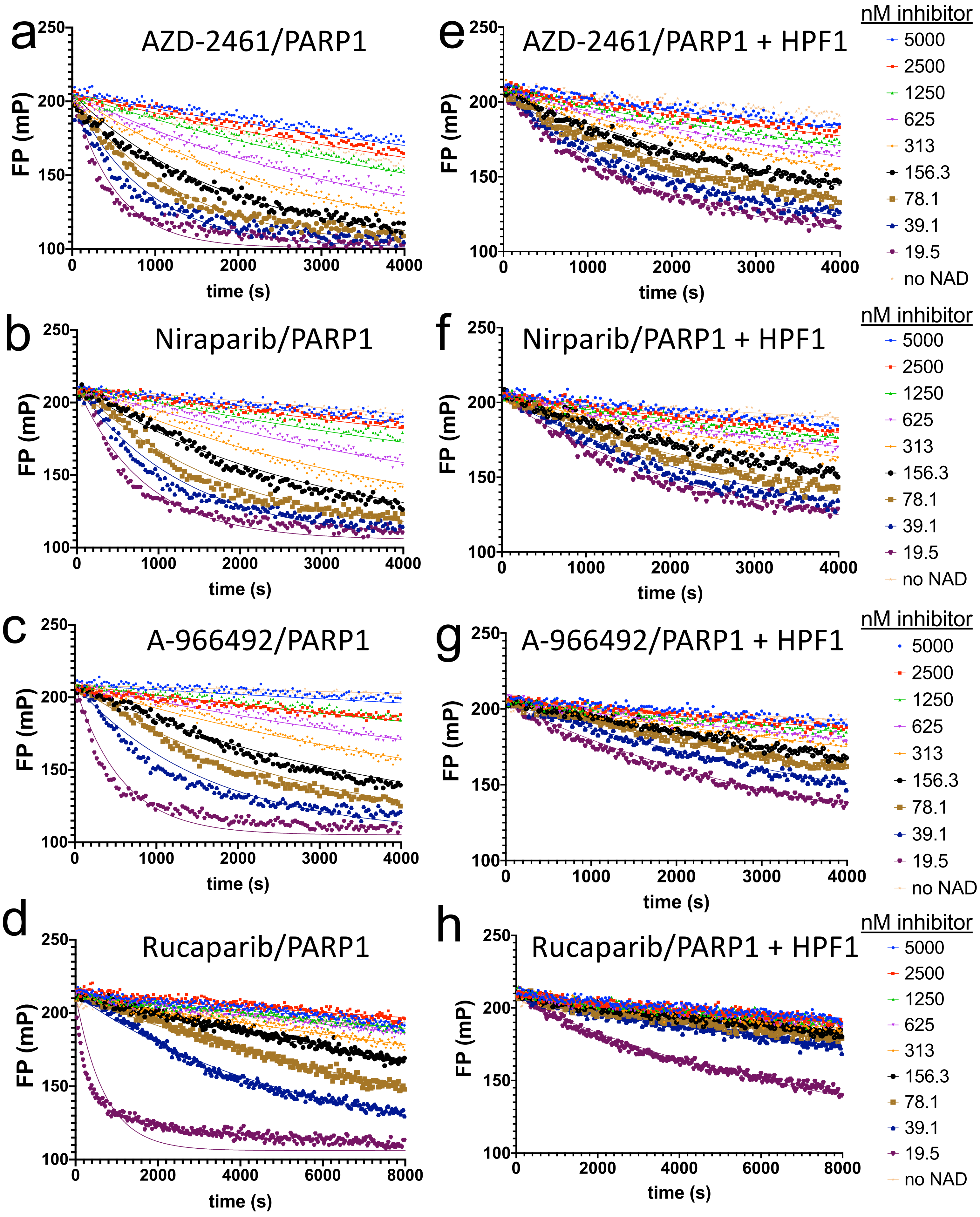
a
AZD-2461/PARP1
e
AZD-2461/PARP1 + HPF1
b
Niraparib/PARP1
f
Nirparib/PARP1 + HPF1
c
A-966492/PARP1
g
A-966492/PARP1 + HPF1
d
Rucaparib/PARP1
h
Rucaparib/PARP1 + HPF1
FP (mP)
time (s)
250
200
150
100
0
1000
2000
3000
4000
4000
6000
8000
nM inhibitor
5000
2500
1250
625
313
156.3
78.1
39.1
19.5
no NAD

Supp. Fig. 2

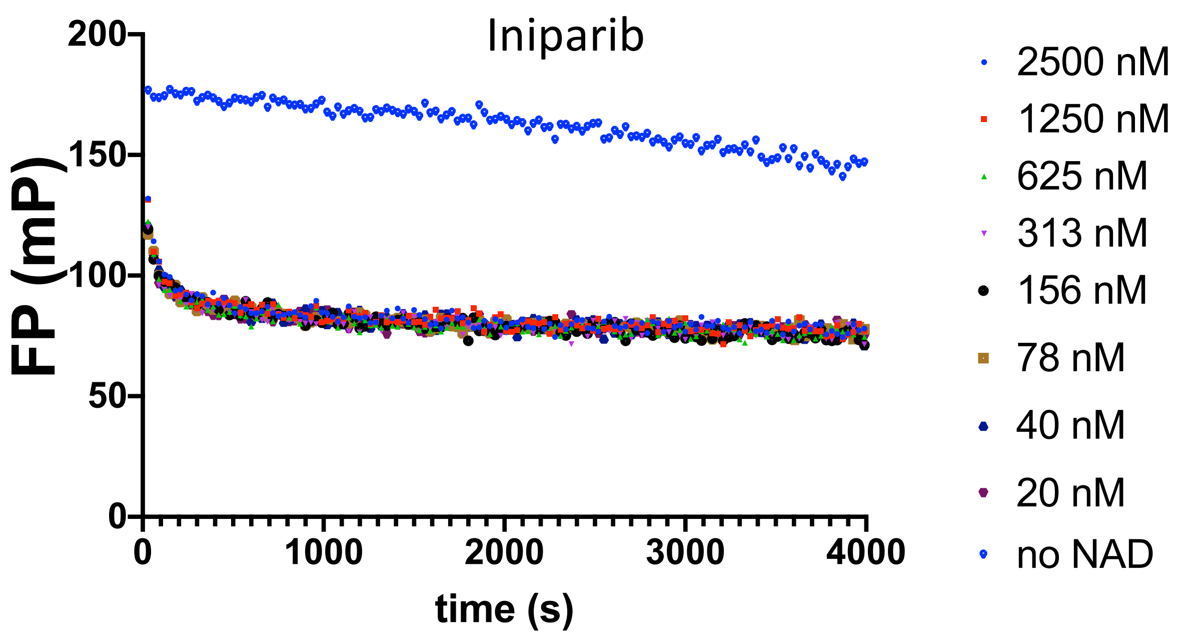
Iniparib
FP (mP)
200
150
100
50
0
0
1000
2000
3000
4000
time (s)
2500 nM
1250 nM
625 nM
313 nM
156 nM
78 nM
40 nM
20 nM
no NAD

Supp Fig. 3

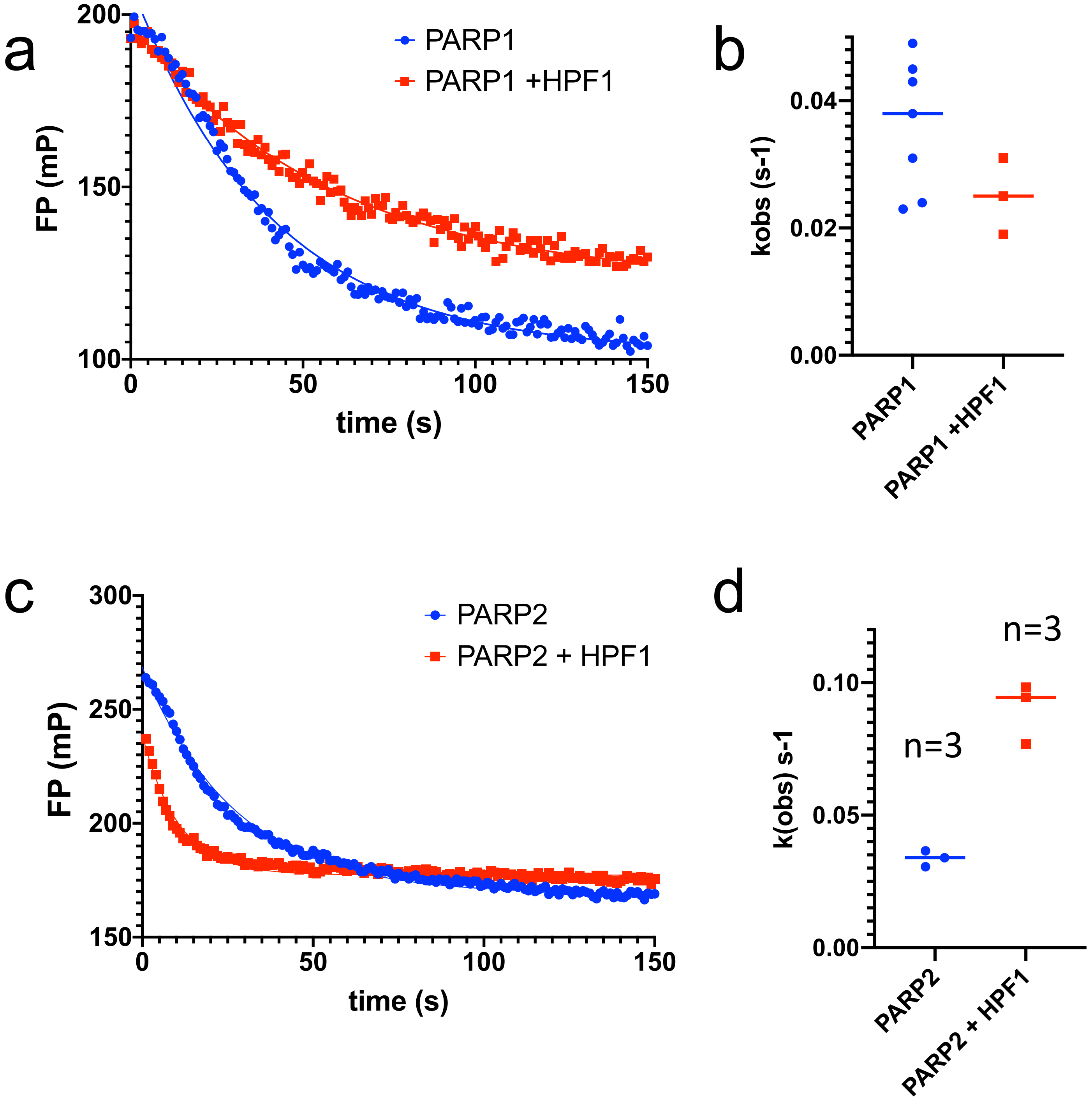
a
200
150
100
0
50
100
150
FP (mP)
time (s)
PARP1
PARP1 +HPF1
b
0.04
0.02
0.00
kobs (s-1)
PARP1
PARP1 +HPF1
c
300
250
200
150
0
50
100
150
FP (mP)
time (s)
PARP2
PARP2 + HPF1
d
n=3
n=3
0.10
0.05
0.00
k(obs) s-1
PARP2
PARP2 + HPF1

Supp Fig. 4

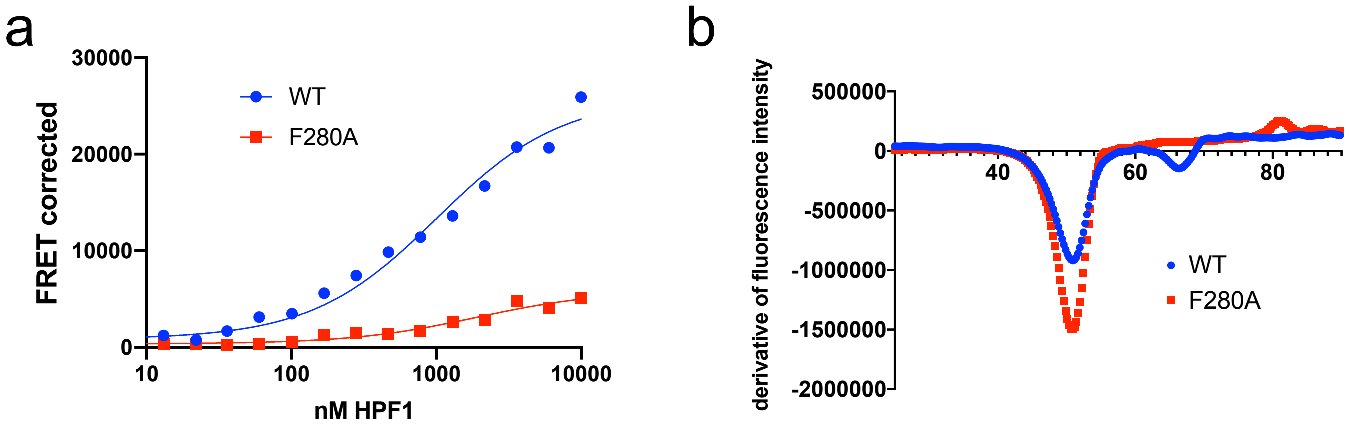

a
30000
20000
10000
0
FRET corrected
WT
F280A
10
100
1000
10000
nM HPF1
b
derivative of fluorescence intensity
500000
0
-500000
-1000000
-1500000
-2000000
40
60
80
WT
F280A

Supp. Fig. 5
