# Supplementary material for: Histone Parylation factor 1 contributes to the inhibition of PARP1 by cancer drugs
Source: Nat Commun. 2021 Feb 2;12:736. doi: 10.1038/s41467-021-20998-8 (PMC7854685; doi:10.1038/s41467-021-20998-8)
Supplement: Supplementary file 2 — Peer Review [file 41467_2021_20998_MOESM2_ESM.pdf]

Reviewer #1 (Remarks to the Author):

This manuscript by the Luger is very novel in its approach. This work analyzes the role of the allosteric HPF1 on PARP-1 with PARP inhibitors.
Of note is the use of NUC 165 DNA to stimulate PARP-1 activity and study its trapping during PARP inhibitor. The use of a longer DNA with double strand breaks is analogous to long DNA and differs from short oligonucleotide to study PARP-1 activity and binding. In this work the authors propose a new mechanism by which HPF1 could increase PARP trapping when it binds the competitive inhibitors.

These interactions between HPF1, PARP-1 and its inhibitors leads to a multistep process where HPF1 leads to more PARP-1 trapping. However this multistep PARP-1 binding to DNA breaks as been described in a recent paper by the groups of Ben Black and John Pascal published in Science on April third 2020. So the present model should be revisited in the light of this Science paper.

Reviewer #2 (Remarks to the Author):

Histone PARylation Factor 1 Contributes to the Inhibition of PARP1 by Cancer Drugs
Johannes Rudolph1, Genevieve Roberts1, Karolin Luger

This study investigates the influence of HPF1 on PARP1 inhibition. The study primarily uses an assay that measures PARP1 interaction with fluorescently labeled DNA. The addition of substrate NAD+ to a PARP1/DNA complex provides the means for PARP1 to modify itself with ADP-ribose, which ultimately leads to PARP1 releasing from the fluorescently labeled DNA, with a corresponding decrease in the polarization of fluorescence emission. PARP inhibitors block NAD+ binding and ADP-ribose production, and have the capacity to prevent PARP1 release. The authors have developed a way to treat this data to extract an inhibition constant for PARP inhibitors. Their treatment of the data indicates that HPF1 enhances the inhibition capacity of certain PARP inhibitors. Although the data are interesting and address an important topic, the work seems more like the start of a story, rather than a complete new finding. As presented, the work seems more appropriate for a short report, or for publication in a different type of journal.

Since HPF1 alone alters PARP1 release from DNA in their experimental setup, it is not clear that they can confidently attribute the observed effect to a difference in binding affinity. Do they see the same result using SPR, a different approach that has been used for measuring PARP inhibitor binding? The FRET assay that they perform is also indirect. Since HPF1 alone influences the release rate, this indicates that the two reactions, PARP1 alone and PARP1/HPF1, are undergoing different processes of release. It could be that HPF1 itself actually slows the PARP1 reaction, and this is indeed predicted based on the biochemical data from the Ahel laboratory. But there could also be an effect that PARP1 modification on Ser residues in the presence of HPF1, versus modification on Glu residues in the absence of HPF1, leads to different mechanisms of PARP1 release from DNA. The authors state that their treatment of the data deconvolutes the effect of HPF1 alone versus PARP inhibitor effects, but it is not clear to me how this could be. This enforces that a direct measurement of PARP inhibitor binding affinity is warranted, such as SPR.

Reviewer #3 (Remarks to the Author):

The manuscript by Rudolph et al. (Luger team) reports how the release of fluorescently labeled DNA from the NAD-dependent enzyme and poly-ADP-ribose polymerase PARP1 is affected by PARP inhibitors and the presence of the histone PARylation factor HPF1, a protein that is key to PARP1 and PARP2’s role in the DNA damage response. Using this in vitro FRET-based assay, they show that HPF1 increases the affinity for a PARP1 – DNA complex of the PARPi Olaparib by about 5-fold, whereas other PARPi such as veliparib and the most potent PARPi talazoparib do not impact the in vitro release

kinetics. Interestingly, this is also seen for the release of a PARP1-nucleosome complex, a likely more physiological template for PARP1 under DNA damage conditions. In order to help rationalize these findings, the authors used docking of the inhbitors into a model of the PARP1-HPF1 complex structure, based on the pioneering structural work of the Ahel team on PARP2-HPF1.

This identified a potential pi-pi-pi stack involving Phe280 of HPF1, Tyr907 of PARP1 and the aromatic ring structure of Olaparib, which is not seen for veliparib, for example. The authors begin and finish the manuscript by suggesting that improved PARP inhibitor design should take into account the HPF1 complex with PARP1.

The biophysical data presented here, which appears thoughtfully executed, show that the HPF1 protein impacts the dissociation of Olaparib-bound PARP1 from DNA, with little imopact on veliparib (for example). This indicates that HPF1 does modulate PARP1's interaction with DNA in the context of bound inhibitors. This novel finding establishes what the high-resolution HPF1-PARP2 structure predicted and, as such, is worth reporting. However, whether the observed 5-fold differences are enough to make a difference and to account for a strategy toward improved PARPi design is not addressed sufficiently well and is also not clear based on what is presented. PARP2 and PARP3 enzymes may also potentially account for some of the anticancer benefits of PARP inhibitors. Also, some of the most potent PARP1/2/3 inhibitors in vitro and in cancer cells, e.g. talazoparib, are barely affected by HPF1. This ought to be commented on, as it risks undermining the rationale for the study and the impact of the current experimental evidence toward new inhibitor designs.

Moreover, there is significant debate whether and how so-called "PARP trapping", a quite controversial term in the field, contributes to PARP enzyme function and the clinical efficacy of one vs. another PARP inhibitor. Since measuring release kinetics by FRET may bear some limited semblance to measuring PARP release kinetics and thus be a proxy for "PARP trapping", the authors may want to add a line or two on that.

In sum: potentially interesting observations for the impact of Hpf1 on the PARP inhibitor Olaparib for PARP1 function. Would strongly recommend adding PARP2 in the analysis and extending the analysis of the PARPi effects on PARP1 in living cells, both wild-type and Hpf1-deleted cells.

Experimental suggestions:

(1)
My main suggestion reflect the impact of distinct PARPi on the release mechanisms of PARP2 vis-à-vis PARP1, which ought to be tested. In all likelihood, PARP inhibitor potency in cancer settings also involves PARP2, so I think that a comparative study with PARP2 would really strengthen the impact of this manuscript. In the absence of this analysis, I am not so convicned that a 5-fold change in PARP1-HFP-1-nucleosome interactions necessarily provide a basis for a renewed effort toward more selective PARP1 inhibitors.

(2)
Ideally, the strong biophysical data in vitro would be complemented by measuring the effects of distinct PARP inhibitors on the recruitment and release kinetics of PARP1 from DNA damage sites in living cells. This could either be done by using cell-based FRAP assays upon addition of a PARP-activating genotoxic stress or perhaps PARP1 recruitment/dissociation kinetics upon UVA laser-induced DNA microirradation experiments, in both wild-type and HPF1-deleted cells, respectively. Such evidence would strengthen the claims made in this analysis and may even reveal strong than 5-fold differences in a cancer cell context. This or alternative cell-based assays would strengthen the impact of the manuscript considerably and make the manuscript interesting for a much broader audience.

Comments on the data interpretation and impact statements for this work:

While the authors' results do clearly show that there is a lot to be gained from studying how the PARP inhibitors interact with PARP enzymes in the context of their complex with the co-factor HPF1, which is key to DNA damage and repair, the impact of the current study would be strengthened by a complementary analysis of how the distinct PARPi affect also the PARP2-HPF1 complex. While the clinical effect of PARP inhibitors have largely been ascribed to the PARP1 enzyme and also in some contexts to PARPi-induced PARP1 trapping on chromatin, some inhibitors such as Olaparib may actually work through PARP2 in terms of their clinical benefit. An analogous analysis with PARP2 would thus signficiantly strengthen the impact of this manuscript. In the absence of these data, some of the claims should in our opinion be dampened, as they are premature:
- Lines 26 and 27: "Our results have important implications for the interpretation of PARP inhibition by current PARPi as well as for the design and analysis of the next generation of PARPi". At a minimum, this should be corrected to "PARP1 inibition".
- Lines 54: "All clinically relevant PARPi bind in the NAD+ binding pocket of PARP1." Add "PARP1 and PARP2".
- Line 60: "HPF1 is an intimate partner of PARP1". And of PARP2, right?
- Line 66: "Here we demonstrate that HPF1 significantly increases the affinity of some but not all PARPi". Add "for PARP1".
- Discussion, line 230: "Our results in part explain the puzzling differences in efficacy of olaparib, niraparib, rucaparib, and talazoparib, compared to veliparib, as single-use agents in the treatment of BRCA-negative cancers". I would urge removing the word "puzzling". I also would recommend replacing the word "in part explain the puzzling" with "may contribute to the observed".
- Line 264: "and thus for cancer treatment". What is the experimental evidence that would strongly support the notion that a more selectie PARP1 inhibitor might be better for cancer therapy than, for example, a mixed PARP1/2/3 inhibitor or, for example, a selective PARP2 or selective PARP3 inhibitor? My impression is that this evidence is not so robust in the literature. Some of the fpoundational work was even done in cells that lacked endogenous PARP2, such as chicken DT40 cells. Literature supporting the key role of PARP1 vs. PARP2 and/or PARP3 should be clearly cited and discussed, prior to making the claim that a more selective PARP1 inhibitor would be better for cancer treatment.

Minor comments:

As a fan of structure-led analysis, I can sympathize with the beauty of the observed pi-pi-pi stack involving Phe280 of HPF1, Tyr907 of PARP1 and the aromatic ring of Olaparib. However, I would remove the word "beautiful" in line 246 of the MS. While docking is powerful and useful, the results of the docking do not replace experimentally determined high-resolution structures. Interpretations should thus be made with caution and neutral language.

Similar point: on line 101, the authors state "As a control, we found that the infamous compound iniparib, whose ability to inhibit PARP1 was later debunked, does not have this effect". I think we can all agree that this molecule is not a PARP inhibitor and that its development and "successful" commercialization/sale to Sanofi exemplifies bad science. However, I would urge removing the word "infamous" and replacing "ability to inhibit PARP1 was later debunked" with "reported ability to inhibit PARP1 was not confirmed by later studies". I think a bibliographic reference or two would also be warranted.

Reviewer response
We thank the reviewers for their detailed and constructive comments on our submission entitled "Histone PARylation Factor 1 Contributes to the Inhibition of PARP1 by Cancer Drugs". Based on their feedback, we have performed numerous additional experiments and significantly revised the manuscript, as described in detail below.
To summarize, we have added a new main Figure 4 and Table 2 to show an orthogonal approach that demonstrates the HPF1-effect on the binding of olaparib to PARP1. We have also incorporated a series of experiments with PARP2, as suggested by reviewer 3. We have added several new columns of data to Table 1 and Supp. Table 1 as well as a new Supp. Fig. 1 and a modified Supp Fig. 4 (C, D) that describe the results obtained for PARP2 in the presence or absence of HPF1. Additionally, we have validated the PARP2 results using the same orthogonal approach as for PARP1 as shown in Figure 4C,D. We have also added two more panels to Figure 5 (C,D) to expand the discussion of the results in the context of the structures. Changes in the manuscript are in blue.
We hope that with this addition of a large body of new data, and with the incorporation of editorial changes which together addresses the vast majority of the reviewer's concerns, you will now find this manuscript suitable for publication.

REVIEWER COMMENTS

Reviewer #1 (Remarks to the Author):

This manuscript by the Luger is very novel in its approach. This work analyzes
the role of the allosteric HPF1 on PARP-1 with PARP inhibitors.
Of note is the use of NUC 165 DNA to stimulate PARP-1 activity and study its trapping during PARP inhibitor. The use of a longer DNA with double strand breaks is analogous to long DNA and differs from short oligonucleotide to study PARP-1 activity and binding. In this work the authors propose a new mechanism by which HPF1 could increase PARP trapping when it binds the competitive inhibitors.

These interactions between HPF1, PARP-1 and its inhibitors leads to a multistep process where HPF1 leads to more PARP-1 trapping. However this multistep PARP-1 binding to DNA breaks as been described in a recent paper by the groups of Ben Black and John Pascal published in Science on April third 2020. So the present model should be revisited in the light of this Science paper.
We have added a paragraph to the Introduction about synthetic lethality, PARP-trapping, and PARP allostery that includes a reference to the recent work by Black and Pascal. To be noted, the paper by Pascal and colleagues describe a very interesting phenomenon that however does not address the role of HPF1 in the binding of PARPi to PARP1.

Reviewer #2 (Remarks to the Author):

Histone PARylation Factor 1 Contributes to the Inhibition of PARP1 by Cancer Drugs
Johannes Rudolph1, Genevieve Roberts1, Karolin Luger

This study investigates the influence of HPF1 on PARP1 inhibition. The study primarily uses an assay that measures PARP1 interaction with fluorescently labeled DNA. The addition of substrate NAD+ to a PARP1/DNA complex provides the means for PARP1 to modify itself with ADP-ribose, which ultimately leads to PARP1 releasing from the fluorescently labeled DNA, with a corresponding decrease in the polarization of fluorescence emission. PARP inhibitors block

NAD+ binding and ADP-ribose production, and have the capacity to prevent PARP1 release. The authors have developed a way to treat this data to extract an inhibition constant for PARP inhibitors. Their treatment of the data indicates that HPF1 enhances the inhibition capacity of certain PARP inhibitors. Although the data are interesting and address an important topic, the work seems more like the start of a story, rather than a complete new finding. As presented, the work seems more appropriate for a short report, or for publication in a different type of journal.
We respectfully disagree. We present a new phenomenon that has not been previously investigated, with important implications for the design of more specific and efficient PARP inhibitors. Because of the importance of PARPi for the treatment of BRCA-deficient patients, any progress in understanding the mechanism or the route to better inhibition needs to be communicated rapidly to the field so that others who are so equipped (i.e. drug development divisions in Pharma) can begin their efforts to generate the next generation of PARPi.

Since HPF1 alone alters PARP1 release from DNA in their experimental setup, it is not clear that they can confidently attribute the observed effect to a difference in binding affinity.
The changed rate of release of DNA due to just HPF1 addition was taken into account in our analysis. See Supp. Fig. 4 and the derivation on pages 10 – 11.
Do they see the same result using SPR, a different approach that has been used for measuring PARP inhibitor binding?
Based on the reviewer's suggestion, we invested some time to use SPR to measure the binding affinity of PARPi for PARP1 directly. Using the published protocols in ref 19 and ref 20, we were able to attach PARP1 to SPR chips. However, we did not observe an SPR signal upon addition of DNA, a worrisome finding given the high affinity of PARP1 for all types of DNA. Thus, we are concerned that the integrity of bound PARP1 might be compromised, and we are thus uncertain what to make of the signal changes we do observe upon addition of PARPi. (Note that DNA binding is required to open up the active site of PARP1). For these reasons, we decided against including these data in the revised manuscript.
The FRET assay that they perform is also indirect.
1) The measurement of inhibition of DNA-release by PARPi is a direct read-out of the activity of PARP1. 2) Although the FRET assay is indirect, according to the thermodynamic principles discussed on page 6 and in Fig. 3, it does reveal the real effects of HPF1 binding on the affinity of PARPi.
Since HPF1 alone influences the release rate, this indicates that the two reactions, PARP1 alone and PARP1/HPF1, are undergoing different processes of release. It could be that HPF1 itself actually slows the PARP1 reaction, and this is indeed predicted based on the biochemical data from the Ahel laboratory.
We agree that HPF1 modulates the rate of PARylation for PARP1, but we have taken this into account in our analysis (See Supp. Fig. 4 and the derivation on pages 10 -11). To clarify, we have added a few sentences to the Methods section on pg. 10. Please note that the HPF1 effect on PARPi is observed for some, but not all inhibitors. Thus, if we had failed to properly account for the effect of HPF1 on PARP1 in the absence of inhibitors, then all inhibitors should have the same effect, or lack thereof.
But there could also be an effect that PARP1 modification on Ser residues in the presence of HPF1, versus modification on Glu residues in the absence of HPF1, leads to different mechanisms of PARP1 release from DNA.
The mechanism is not important here, only the rate is important for deriving K_Is, and as noted above, we have taken this into account.
The authors state that their treatment of the data deconvolutes the effect of HPF1 alone versus PARP inhibitor
effects, but it is not clear to me how this could be.

See response above.
This enforces that a direct measurement of PARP inhibitor binding affinity is warranted, such as SPR.
We agree with the reviewer in that orthogonal approaches are important for validating novel and unexpected findings. We have therefore developed a method to directly measure the affinity of fluorescently-labeled Olaparib to PARP1 (and PARP2; see below) that agrees remarkably well with our findings for the effect of HPF1 on the binding of unlabeled olaparib. This additional confirmation of our results provides an important check on our methods and our analysis. See the new paragraph on pp 6 -7 and the new Figure 4 and Table 2.

Reviewer #3 (Remarks to the Author):

The manuscript by Rudolph et al. (Luger team) reports how the release of fluorescently labeled DNA from the NAD-dependent enzyme and poly-ADP-ribose polymerase PARP1 is affected by PARP inhibitors and the presence of the histone PARylation factor HPF1, a protein that is key to PARP1 and PARP2's role in the DNA damage response. Using this in vitro FRET-based assay, they show that HPF1 increases the affinity for a PARP1 – DNA complex of the PARPi Olaparib by about 5-fold, whereas other PARPi such as veliparib and the most potent PARPi talazoparib do not impact the in vitro release kinetics. Interestingly, this is also seen for the release of a PARP1-nucleosome complex, a likely more physiological template for PARP1 under DNA damage conditions. In order to help rationalize these findings, the authors used docking of the inhbitors into a model of the PARP1-HPF1 complex structure, based on the pioneering structural work of the Ahel team on PARP2-HPF1.

This identified a potential pi-pi-pi stack involving Phe280 of HPF1, Tyr907 of PARP1 and the aromatic ring structure of Olaparib, which is not seen for veliparib, for example. The authors begin and finish the manuscript by suggesting that improved PARP inhibitor design should take into account the HPF1 complex with PARP1.

The biophysical data presented here, which appears thoughtfully executed, show that the HPF1 protein impacts the dissociation of Olaparib-bound PARP1 from DNA, with little imopact on veliparib (for example). This indicates that HPF1 does modulate PARP1's interaction with DNA in the context of bound inhibitors. This novel finding establishes what the high-resolution HPF1-PARP2 structure predicted and, as such, is worth reporting. However, whether the observed 5-fold differences are enough to make a difference and to account for a strategy toward improved PARPi design is not addressed sufficiently well and is also not clear based on what is presented.
We agree that a 5-fold difference may not be sufficient to account for physiological effects. However, note that all existing PARPi were developed without consideration of HPF1, and the point of our manuscript is to reveal that targeting the PARP1 – HPF1 complex instead of PARP1 alone should be considered in further drug development efforts.
PARP2 and PARP3 enzymes may also potentially account for some of the anticancer benefits of PARP inhibitors.
This is most likely true to some extent, although deletion of just PARP1 from cells leads to a significant loss of efficacy of PARPi (see ref. 18).
Also, some of the most potent PARP1/2/3 inhibitors in vitro and in cancer cells, e.g. talazoparib, are barely affected by HPF1. This ought to be commented on, as it risks undermining the rationale for the study and the impact of the current experimental evidence toward new inhibitor designs.

As noted on pg. 5 (second paragraph in Results section with HPF1), our method may not have the needed sensitivity to distinguish whether the binding of talazoparib is affected by HPF1. We have added an additional sentence to the Discussion stating this again so as to avoid any confusion.

Moreover, there is significant debate whether and how so-called “PARP trapping”, a quite controversial term in the field, contributes to PARP enzyme function and the clinical efficacy of one vs. another PARP inhibitor. Since measuring release kinetics by FRET may bear some limited semblance to measuring PARP release kinetics and thus be a proxy for “PARP trapping”, the authors may want to add a line or two on that.
The reviewer makes an important point about the complexity of PARP-trapping. We have purposely avoided bringing this topic into our Discussion, as the effect we observe here does not have anything to do with the phenomenon of PARP-trapping, and we do not wish to add further confusion to this field. To emphasize this point, we have added a brief comment in the Discussion.

In sum: potentially interesting observations for the impact of Hpf1 on the PARP inhibitor Olaparib for PARP1 function. Would strongly recommend adding PARP2 in the analysis and extending the analysis of the PARPi effects on PARP1 in living cells, both wild-type and Hpf1-deleted cells.
This was added – see below.

Experimental suggestions:

(1)
My main suggestion reflect the impact of distinct PARPi on the release mechanisms of PARP2 vis-à-vis PARP1, which ought to be tested. In all likelihood, PARP inhibitor potency in cancer settings also involves PARP2, so I think that a comparative study with PARP2 would really strengthen the impact of this manuscript. In the absence of this analysis, I am not so convicned that a 5-fold change in PARP1-HFP-1-nucleosome interactions necessarily provide a basis for a renewed effort toward more selective PARP1 inhibitors.
As suggested by the reviewer, we have now performed these assays for PARP2. See the significant additions to the text, the new Supp. Fig. 1. and Supp. Fig 4C,D, and the additional columns in both Table 1 and Supp. Table 1. The results are quite interesting, in that PARP2 does not show the same HPF1-effect as PARP1. We thank the reviewer for their encouragement to explore this further and think it is very interesting that the HPF1-effect does not translate to PARP2.

(2)
Ideally, the strong biophysical data in vitro would be complemented by measuring the effects of distinct PARP inhibitors on the recruitment and release kinetics of PARP1 from DNA damage sites in living cells. This could either be done by using cell-based FRAP assays upon addition of a PARP-activating genotoxic stress or perhaps PARP1 recruitment/dissociation kinetics upon UVA laser-induced DNA microirradiation experiments, in both wild-type and HPF1-deleted cells, respectively. Such evidence would strengthen the claims made in this analysis and may even reveal strong than 5-fold differences in a cancer cell context. This or alternative cell-based assays would strengthen the impact of the manuscript considerably and make the manuscript interesting for a much broader audience.
We have made several attempts to address this reviewer’s excellent suggestions about complementing our in vitro data using cell-based assays. We have used UVA laser-induced DNA micro-irradiation experiments as previously described (doi.org/10.1016/j.bpj.2019.04.032).

We find no change in the accumulation of PARP1 at sites of DNA damage in response to PARPi (unpublished data), as expected and as also recently described by doi.org/10.1093/nar/gkaa718. In our experiments using overexpressed PARP1, we do not see significant release of PARP1 from sites of DNA damage prior to extensive photobleaching, and thus we are unable to evaluate the effect of PARPi on this process. Although we have recently developed a CRISPR cell line with labeled PARP1 that allows for measurement of PARP1 departure from sites of DNA damage, producing such a cell line in an HPF1-/- background is beyond the scope of this manuscript. We also note that the Ahel group has observed that HPF1-/- are particularly sensitive to PARPi, as if they become BRCA-negative and therefore exhibit synthetic lethality (ref 22). This complication of deleting HPF1 suggests that unknown effects may greatly complicate the interpretation of in vivo data, and this would require more extensive investigation that we believe is beyond the scope of the current manuscript

Comments on the data interpretation and impact statements for this work:

While the authors' results do clearly show that there is a lot to be gained from studying how the PARP inhibitors interact with PARP enzymes in the context of their complex with the co-factor HPF1, which is key to DNA damage and repair, the impact of the current study would be strengthened by a complementary analysis of how the distinct PARPi affect also the PARP2-HPF1 complex.
Done, as requested.

While the clinical effect of PARP inhibitors have largely been ascribed to the PARP1 enzyme and also in some contexts to PARPi-induced PARP1 trapping on chromatin, some inhibitors such as Olaparib may actually work through PARP2 in terms of their clinical benefit. An analogous analysis with PARP2 would thus signficiantly strengthen the impact of this manuscript.
Done, as requested.
In the absence of these data, some of the claims should in our opinion be dampened, as they are premature:
- Lines 26 and 27: "Our results have important implications for the interpretation of PARP inhibition by current PARPi as well as for the design and analysis of the next generation of PARPi". At a minimum, this should be corrected to "PARP1 inibition".
As we have now also analyzed inhibition by PARP2, the statement was not modified. The affinities of some currently existing PARPi to PARP1, but not PARP2, are modulated by HPF1, a finding that is an important consideration for the design of the next generation of PARPi.
- Lines 54: "All clinically relevant PARPi bind in the NAD+ binding pocket of PARP1." Add "PARP1 and PARP2".
Done, as requested.
- Line 60: "HPF1 is an intimate partner of PARP1". And of PARP2, right?
Correct. Fixed.
- Line 66: "Here we demonstrate that HPF1 significantly increases the affinity of some but not all PARPi". Add "for PARP1".
We have added the new results with respect to PARP2 into this sentence.
- Discussion, line 230: "Our results in part explain the puzzling differences in efficacy of olaparib, niraparib, rucaparib, and talazoparib, compared to veliparib, as single-use agents in the treatment of BRCA-negative cancers". I would urge removing the word "puzzling". I also would recommend replacing the word "in part explain the puzzling" with "may contribute to the observed".
Done, as requested.
- Line 264: "and thus for cancer treatment". What is the experimental evidence that would

strongly support the notion that a more selectie PARP1 inhibitor might be better for cancer therapy than, for example, a mixed PARP1/2/3 inhibitor or, for example, a selective PARP2 or selective PARP3 inhibitor? My impression is that this evidence is not so robust in the literature. Some of the fpoundational work was even done in cells that lacked endogenous PARP2, such as chicken DT40 cells. Literature supporting the key role of PARP1 vs. PARP2 and/or PARP3 should be clearly cited and discussed, prior to making the claim that a more selective PARP1 inhibitor would be better for cancer treatment.
The strongest evidence for PARP1 being the current primary target of PARPi comes from studies that demonstrate deletion of PARP1 greatly reduces the sensitivity of cells to PARPi (e.g. see ref 18). Given the role of HPF1 in the DNA damage response, we are merely proposing that efforts should be made to target the PARP1 – HPF1 complex, not just free PARP1.

Minor comments:

As a fan of structure-led analysis, I can sympathize with the beauty of the observed pi-pi-pi stack involving Phe280 of HPF1, Tyr907 of PARP1 and the aromatic ring of Olaparib. However, I would remove the word “beautiful” in line 246 of the MS. While docking is powerful and useful, the results of the docking do not replace experimentally determined high-resolution structures. Interpretations should thus be made with caution and neutral language.
Done, as requested.

Similar point: on line 101, the authors state “As a control, we found that the infamous compound iniparib, whose ability to inhibit PARP1 was later debunked, does not have this effect”. I think we can all agree that this molecule is not a PARP inhibitor and that its development and “successful” commercialization/sale to Sanofi exemplifies bad science. However, I would urge removing the word “infamous” and replacing “ability to inhibit PARP1 was later debunked” with “reported ability to inhibit PARP1 was not confirmed by later studies”. I think a bibliographic reference or two would also be warranted.
Done, as requested.

Reviewer #2 (Remarks to the Author):

The addition of the fl-olaparib binding experiments, and the clarification/additional text regarding the model and fitting for KI values, were nice additions to the study.

I did not find the suggested "lysine lock" very compelling for describing the difference between PARP1 and PARP2 in terms of olaparib/HPF1 effects. It seems the lysine will just sample the two conformations shown, and the crystal structure just highlighted one of them. Otherwise, what makes the lysine adopt one conformation over the other?

Other points:
* legend issue
The labeling in Figure 4B is not clear for Nuc165, the legend says read line with blue dot, but I do not see that in the plot (only the blue dots, and it took me some time to figure this out).

* methods typo, I believe
signal was monitored every minute for 10,000 min. (that's about a week!!

* type
since HPF1-mdeiated PARylation

Reviewer #3 (Remarks to the Author):

The revised manuscript by Rudolph et al. (Luger team) brings in substantial new results that add to this manuscript, especially for the impact of HPF1 and PARPi on PARP2, which this reviewer solicited. These data reveal interesting, opposite effects of HPF1 on PARP1 and PARP2 with regard to their interaction with distinct PARPi molecules. Further, a good attempt is made at rationalizing these distinct behaviours based on the similar, but distinct interactions of Olaparib with the active site of both enzymes. The new biophysical data added for PARP2 truly add to the manuscript and broaden the impact of the study.

My other comment regarding PARP trapping is addressed appropriately by the authors and a discussion of this is beyond the scope of this manuscript now that additional data for PARP2 have been added.

Regarding my apparent insistence on asking the authors to be cautious on their interpretation of PARP1's primary role as the driver of the efficacy of PARP inhibitors, the authors twice replied that "deletion of just PARP1 from cells leads to a significant loss of efficacy of PARPi" and refer to reference 18, a publication by Yves Pommier that used chicken DT40 cells, as I previously wrote. The problem, I think, here is that the avian genome lacks a PARP2 gene. Chickens have no PARP2. Avian DT40 lymphoblast PARP1-/- cells are considered to be functionally equivalent to a mammalian PARP1 and PARP2 double knockout cell, which is one reason why they have been so popular to use. So while a PARP1 KO in DT40 cells ablates PARPi sensitivity (showing that PARP1 causes PARPi sensitivity in CHICKEN cells), this does not mean that PARP1 is necessarily the primary target of PARPi sensitivity in mammalian cells. Evidence for this is actually more scant than one might generally assume. In other words: a mammalian PARP1 knockout cell line is not functionally equivalent to a chicken DT40 PARP1-/- cell. PARP1 is the primary target of PARPi in DT40 cells, because PARP1 is potentially the ONLY DNA-damage relevant target of PARPi in chickens. The Pommier paper is not the only one using the very powerful DT40 cell model in the history of PARP1 and PARPi. I simply think that we may need to be a bit more careful in mammals and treat many interpretations derived from chicken DT40 cells with "a grain of salt". PARPi may work through more than just PARP1 in mammals, including PARP3 and others.
All other minor points are well addressed in the excellently revised manuscript.

REVIEWERS' COMMENTS

Reviewer #2 (Remarks to the Author):

The addition of the fl-olaparib binding experiments, and the clarification/additional text regarding the model and fitting for KI values, were nice additions to the study.

I did not find the suggested "lysine lock" very compelling for describing the difference between PARP1 and PARP2 in terms of olaparib/HPF1 effects. It seems the lysine will just sample the two conformations shown, and the crystal structure just highlighted one of them. Otherwise, what makes the lysine adopt one conformation over the other?
Since we saw no other possible explanation, and this is the Discussion section of the paper, we prefer to provide some hint to the reader of what to look for rather than just saying nothing.

Other points:

* legend issue
The labeling in Figure 4B is not clear for Nuc165, the legend says read line with blue dot, but I do not see that in the plot (only the blue dots, and it took me some time to figure this out).
We apologize for the confusion and thank the reviewer for noticing. Fixed.

* methods typo, I believe
signal was monitored every minute for 10,000 min. (that's about a week!!?)
Fixed; it should have said 1000 min.

* type
since HPF1-mdeiated PARylation
Fixed.

Reviewer #3 (Remarks to the Author):

The revised manuscript by Rudolph et al. (Luger team) brings in substantial new results that add to this manuscript, especially for the impact of HPF1 and PARPi on PARP2, which this reviewer solicited. These data reveal interesting, opposite effects of HPF1 on PARP1 and PARP2 with regard to their interaction with distinct PARPi molecules. Further, a good attempt is made at rationalizing these distinct behaviours based on the similar, but distinct interactions of Olaparib with the active site of both enzymes. The new biophysical data added for PARP2 truly add to the manuscript and broaden the impact of the study.

My other comment regarding PARP trapping is addressed appropriately by the authors and a discussion of this is beyond the scope of this manuscript now that additional data for PARP2 have been added.

Regarding my apparent insistence on asking the authors to be cautious on their interpretation of PARP1's primary role as the driver of the efficacy of PARP inhibitors, the authors twice replied that "deletion of just PARP1 from cells leads to a significant loss of efficacy of PARPi" and refer to reference 18, a publication by Yves Pommier that used chicken DT40 cells, as I previously wrote. The problem, I think, here is that the avian genome lacks a PARP2 gene. Chickens have no PARP2. Avian DT40 lymphoblast PARP1-/- cells are considered to be functionally equivalent to a mammalian PARP1 and

PARP2 double knockout cell, which is one reason why they have been so popular to use. So while a PARP1 KO in DT40 cells ablates PARPi sensitivity (showing that PARP1 causes PARPi sensitivity in CHICKEN cells), this does not mean that PARP1 is necessarily the primary target of PARPi sensitivity in mammalian cells. Evidence for this is actually more scant than one might generally assume. In other words: a mammalian PARP1 knockout cell line is not functionally equivalent to a chicken DT40 PARP1-/- cell. PARP1 is the primary target of PARPi in DT40 cells, because PARP1 is potentially the ONLY DNA-damage relevant target of PARPi in chickens. The Pommier paper is not the only one using the very powerful DT40 cell model in the history of PARP1 and PARPi. I simply think that we may need to be a bit more careful in mammals and treat many interpretations derived from chicken DT40 cells with “a grain of salt”. PARPi may work through more than just PARP1 in mammals, including PARP3 and others.
We have added two references showing that the effect of deletion of PARP1 seen in avian cells (loss of efficacy of PARPi) extends to human cells. See ref. 22,23.

All other minor points are well addressed in the excellently revised manuscript.
